# Protein-RNA Complexes and Efficient Automatic Docking: Expanding RosettaDock Possibilities

**DOI:** 10.1371/journal.pone.0108928

**Published:** 2014-09-30

**Authors:** Adrien Guilhot-Gaudeffroy, Christine Froidevaux, Jérôme Azé, Julie Bernauer

**Affiliations:** 1 AMIB Project, Inria Saclay-Île de France, Palaiseau, France; 2 Laboratoire de Recherche en Informatique (LRI), CNRS UMR 8623, Université Paris-Sud, Orsay, France; 3 Laboratoire d'Informatique de l'École Polytechnique (LIX), CNRS UMR 7161, École Polytechnique, Palaiseau, France; 4 Laboratoire d'Informatique de Robotique et de Microélectronique de Montpellier (LIRMM), CNRS UMR 5506, Université Montpellier 2, Montpellier, France; National Chiao Tung University, Taiwan

## Abstract

Protein-RNA complexes provide a wide range of essential functions in the cell. Their atomic experimental structure solving, despite essential to the understanding of these functions, is often difficult and expensive. Docking approaches that have been developed for proteins are often challenging to adapt for RNA because of its inherent flexibility and the structural data available being relatively scarce. In this study we adapted the RosettaDock protocol for protein-RNA complexes both at the nucleotide and atomic levels. Using a genetic algorithm-based strategy, and a non-redundant protein-RNA dataset, we derived a RosettaDock scoring scheme able not only to discriminate but also score efficiently docking decoys. The approach proved to be both efficient and robust for generating and identifying suitable structures when applied to two protein-RNA docking benchmarks in both bound and unbound settings. It also compares well to existing strategies. This is the first approach that currently offers a multi-level optimized scoring approach integrated in a full docking suite, leading the way to adaptive fully flexible strategies.

## Introduction

Protein-RNA interactions often play a major role in the cell. They are involved in many processes such as replication, mRNA transcription or regulation of RNA levels and control the operation of key cellular machineries such as the RNA induced silencing complex (RISC). They are thus good candidates for therapeutic studies [1]. The variety of proteins able to bind RNA molecule is very large and covers a wide range of protein domains. This includes domains such as RRM and dsRDB which all show RNA binding activity and are well studied [2]. In the recent years, experimental techniques have shed the light on RNA and protein-RNA complexes. X-ray Crystallography [3] and NMR [4], [5] have provided high-resolution structures offering insights into RNA function and binding activity and modes [6], [7] but other experimental techniques have also allowed for the analysis of larger ensembles [8]–[10]. Single-molecule experiments can now provide high-resolution data [11] and the engineering of RNA binding molecule is with reach [12]. Despite the wide interest and advances in structural biology for RNA and protein-RNA complexes, the number of structures available in the PDB is relatively small (a few thousand for RNA molecules and around a thousand for protein-RNA complexes). And both the modelling and the prediction of protein-RNA interactions remain a challenge [13].

The structural modelling of large biomolecules and their interactions is a challenging task. A large number of methods for both predicting and evaluating the results have been developed [14]–[16] and the Critical Assessment of PRediction of Interactions (CAPRI http://capri.ebi.ac.uk) challenge [17] which allowed for an international blind prediction setting has shown that despite great progress, the methods available still rely on a great variety of biological data to be available [18] and the flexibility of the molecules remain a modelling and computational issue to overcome [19]. The techniques are however now able to integrate more data and predict better ion and water molecules which mediate the binding [20]. Binding affinity is not yet a predictable quantity but the originality and first results of the latest strategies is encouraging [21].

Protein-RNA complexes are especially difficult to predict and model for two reasons: the inherent flexibility of RNA molecules and the electrostatics driving the binding as the RNA molecule is negatively charged. Progress in RNA structure prediction and folding [22]–[26] allows to deal with flexibility but have yet to be fully multi-scale [27] and integrated in the docking processes. This can be done once the scoring function for protein-RNA are efficient enough and provide accurate conformation selection. Specially designed coarse-grained force-fields based on statistics [28]–[32] have shown great promises and coarse-grained versions for reducing the initial exploration phase of coarse-grained search are interesting [33], [34]. The optimization is however often based on relatively simple statistics measurements and rarely benefits from the variety of structural datasets recently made available to the community. The Protein-RNA interface database [35] offers high quality curated datasets for statistical analysis. Both available in a redundant and non-redundant version it allows for fine measurements on high-resolution structures. The three protein-RNA benchmarks available in the literature [36]–[38] also offer a great opportunity to assess and review high-resolution structures and predictions.

The availability of structural data is essential for machine learning based strategies for scoring in docking experiments. Various machine learning strategies have been developed in the past for protein-protein complexes [39]–[43] and have proven to be key in reranking and optimizing docking experiments for protein-protein complexes as the last CAPRI rounds has shown [44], [45]. In this study, we use a machine-learning based strategy to optimize the well-known RosettaDock scoring function for high-resolution docking. RosettaDock is a leading edge protein-docking suite [46]–[48] which while being very versatile and widely used have been only seldom used for protein-RNA docking [28], [49]. We first extended the RosettaDock low resolution model to RNA for both searching and scoring. We then used the Protein Interface Database [35] as reference dataset to generated near-native and plausible docking conformations. We then optimized the RosettaDock high-resolution scoring function using supervised machine learning. After cross-validation and carefully handling tests, we assessed the obtained protocol on the protein docking benchmarks I and II [36], [38]. We show that the obtained RosettaDock RNA protocol performs better than in the previous attempts [49] in a semi-rigid body approach for both bound and unbound docking and can undoubtedly be used for successful protein-RNA predictions.

## Materials and Methods

### Protein-RNA complexes training and evaluation sets for RosettaDock

Protein-RNA native X-ray structures for learning were downloaded from the Protein-RNA Interface Database (PRIDB) [35]. The non-redundant PRIDB (RB199) contains 199 RNA chains extracted from the PDB in 2010. From the 134 complexes described in this set, we only kept the binary complexes: one protein and one RNA molecule. We also discarded complexes involving the ribosome because of their redundancy and to avoid biasing towards ribosome data but also to avoid computationally expensive procedures. The resulting native structure dataset from the PRIDB is made of 120 complexes (Table S1).

We also used the two protein-RNA benchmarks [36], [38] as a validation set in bound and unbound (protein and RNA when available) settings. Among the 45 complexes contained in the Benchmark I [36], 11 complexes are not found in the PRIDB. Among the 106 complexes from the Benchmark II, we only kept the 76 complexes for which an unbound structure of the protein exists. Among these 76 complexes, 36 cannot be found in the PRIDB. After checking for overlap on the two benchmarks which were obtained using two different strategies, the resulting test set is made of 40 complexes. The list of complexes used in this study can be found in Table S2.

From all the native structures from both the PRIDB and the benchmarks, near-native and decoy conformations are generated using the Rosetta perturbation protocol [47]. For each pdb file, 10,000 perturbation conformations are to be obtained. Among these 10,000, to allow for correct learning, we want 30 near-native conformations whose Irmsd is smaller than 5 Å and 30 decoy conformations whose Irmsd is greater than 8 Å. Irmsd definition is taken from [14] and adapted to protein-RNA complexes by using the RNA backbone P atoms. For that purpose, the amplitude of the translation and the three rotations applied is chosen to follow a normal law of variance 1 and different expectations (small, regular and large). The regular setting is set to 3 Å for the translation and 8° for the rotations, the small (resp. large) setting is set to 1 Å (resp. 9 Å) for the translation and 4° (resp 27°) for the rotations. For each pdb file, the setting chosen is the smallest allowing for enough near-native and decoy conformation generation.

### RosettaDock protocol and scoring functions

The RosettaDock protocol is two-level docking search: low resolution and high resolution. The low resolution stage uses a coarse-grained representation of the partners to quickly sample the search space for candidates. The high resolution stage rebuilds the all-atom partners from the low resolution candidates to perform a refined atomic search possibly including rotamer search and loop optimization.

The low resolution scoring function uses the backbone of the molecule and one centroid per residue [47] and contains five weighted terms:
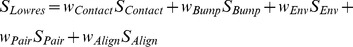
where *S_Contact_* represents the number of interface residues being defined by having a centroid less than 6 Å away from a centroid in the other partner; *S_Bump_* is a distance-based penalty for steric clashes; *S_Env_* defines the probability of finding a residue in a specific environment (buried/exposed and interface/non interface); *S_Pair_* is a pair potential defining the propensity of residues to be found in interaction in given environments and *S_Align_* is an optional term to match a specific alignment pattern (e.g. antibodies).

These five terms of the low resolution score can be computed for protein-RNA complexes in the same way they were for proteins. For RNA, the backbone is chosen to include the sugar ring and the centroid is taken to be the center of mass of the base. All the parameters for the low resolution scoring terms are computed on the PRIDB reference set.

The high resolution scoring function uses all the atoms of the molecules, including the hydrogen atoms, and is made of seven weighted terms:
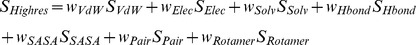
where *S_VdW_* is a Van der Waals term (Lennard-Jones based), *S_Elec_* is a Coulomb term, *S_Solv_* a solvent term based on the Lazaridis-Karplus model, *S_Hbond_* is a H-bond 10–12 potential term, *S_SASA_* is the solvent accessible surface area term (often omitted), *S_Pair_* is a pair potential defining the propensity of residues to be found in interaction in given environments and *S_Rotamer_* is a probability of finding a specific rotamer. Exactly like for the previous low resolution scores, all the terms can be computed for RNA. The rotamer term and loop optimization are switched off for RNA such as in [28] and in previous CAPRI runs containing RNA [49] for which the RosettaDock all-atom procedure was just used to refine the obtained conformation and RNA parameters were derived from protein data.

### Low resolution weights

The low resolution representation for each residue/nucleotide is made of the backbone atoms and one pseudo-atom called centroid to represent the side-chain. For the residues, the location of the centroid is taken from RosettaDock (average over a reference set of PDB structures). For RNA nucleotides, the centroid is taken as the averaged position (See Figure S1). The low resolution scores are computed for RNA on the full PRIDB (more than a thousand structures). They represent counting statistics and are not optimized further.

### High resolution scoring weights optimization strategies

We performed the optimization by supervised learning. To ensure an accurate learning phase, the perturbation was split in two categories for learning labelled near-native (Irmsd<5 Å) and decoy (Lrsmd>8 Å). The assessment was performed using slightly different categories so as to mimic the CAPRI context: near-native (Irmsd<5 Å) and non-native (Irmsd≥5 Å). While these rmsd range are certainly not always likely to accurately represent a correct RNA binding mode, especially considering the variability in size of the RNA molecules, they represent a reachable goal not yet attained by the CAPRI community.

Weights for the all atom scoring function described above were optimized in the [0∶1] interval within the ROC-based Genetic LearneR (ROGER) framework using logistic regression and Receiver Operating Characteristic (ROC) based genetic algorithm as previously described for protein-protein docking [40]. The optimization of the Area Under the ROC curve (ROC-AUC) is performed using 100,000 iterations with μ = 10 and λ = 80.

The first evaluation of the whole scoring procedure is made using cross-validation and a leave-one-*pdb*-out approach. Inspired by the leave-one-out procedure in statistics, we previously used this strategy for machine learning of protein-protein docking scoring functions [40], [41], [43]. For a specific pdb file, all the native, near-native or decoy conformations, that were generated from this file, are removed from the learning set. The evaluation is then performed for this specific pdb file. The original set learning containing 120 complexes, the whole procedure is repeated 120 times. The set being non-redundant, like cross-validation, this computationally expensive process ensures that the result for a specific pdb file is not biased.

**Figure 1:**
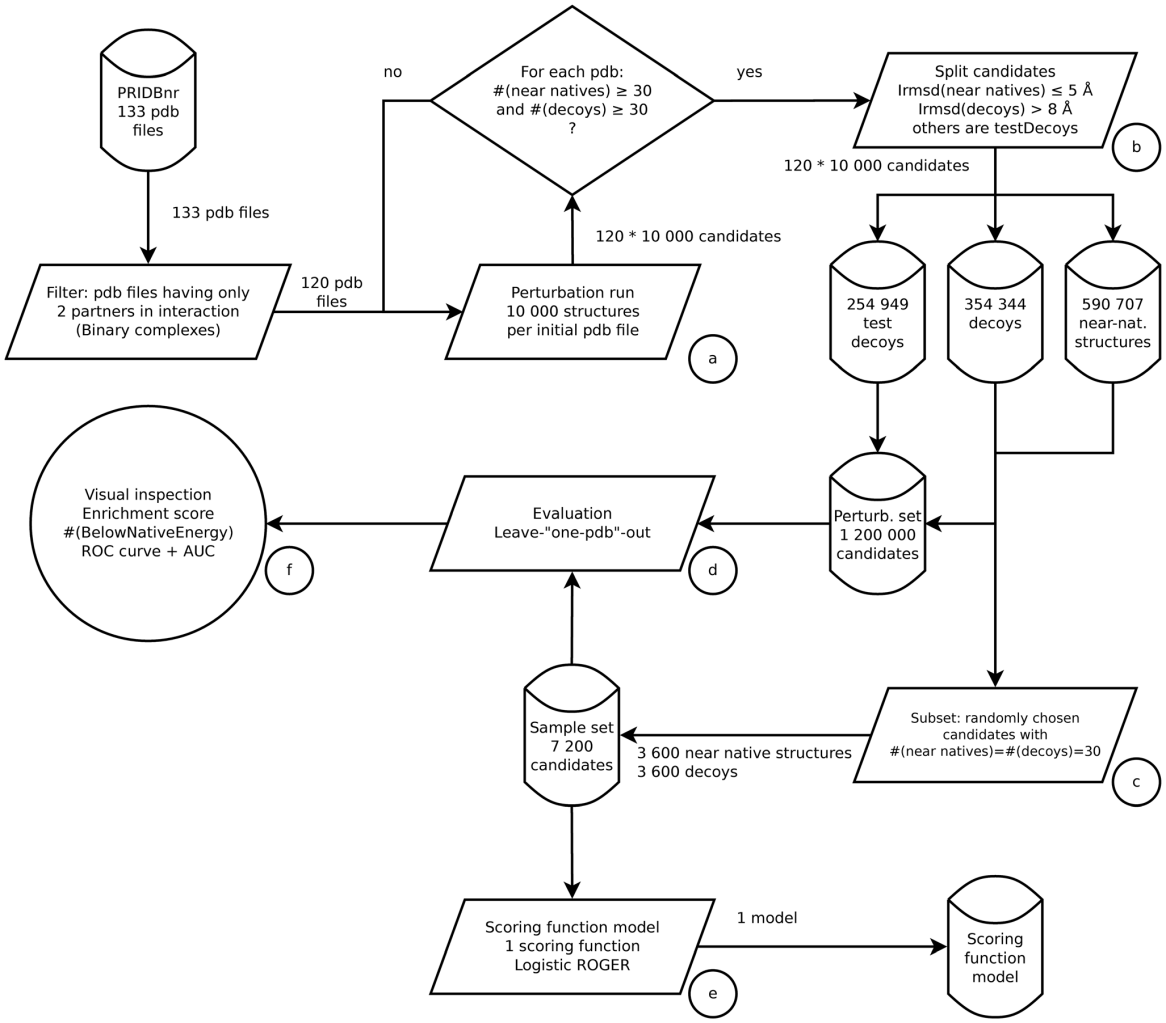
Flowchart of the machine learning strategy. The procedure is made of six steps: a) data processing using the non-redundant PRIDB to generate candidates, b) splitting of candidates into test decoys, decoys, near-native structures according to their Irmsd so as to define the perturbation set, c) definition of the sample set using 30 near-native structures and 30 decoys per native structure - randomly chosen, d) leave-one-pdb-out evaluation, e) scoring function learning using ROGER and f) result analysis.

To also avoid biasing the samples towards a category while learning, learning is performed with 30 near-native and 30 decoy structures for each of the 120 pdb file leading to a total size of 7,200 structures for the learning set (3,600×2). Test is performed on the 10,000 candidates of each test pdb file. The global procedure flowchart is available in Figure 1.

### Assessment

The learning procedure is initially assessed using standard machine learning criteria: analysis of the ROC curve, ROC-AUC in a cross-validation setting and precision for the top 10 structures. CAPRI/Critical Assessment of protein Structure Prediction (CASP) inspired biological criteria are used for the final assessment: Energy vs. Irmsd curve and Enrichment Score (ES). Interface root mean square deviation (Irmsd) is taken from Lensink et al. [50]. We adapted the Enrichment Score from Tsai et al. [51], and also used for RNA structure assessment [52], [53]. The enrichment score is defined as: 
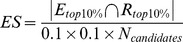
 where *E_top_*_10%_ is the top 10% scoring and *R_top_*_10%_ the best 10% rmsd structures. By looking at the degree of overlap between the two categories, the enrichment score provides insight on how good the scoring is *ES*<1 corresponds to bad scoring, *ES* = 1corresponds to random scoring and *ES* = 10 is perfect scoring. Even if what can be considered good scoring is not obvious, the comparison of *ES* values between 1 and 10 provides good information on how well the strategy performs on different targets.

## Results and Discussion

### Native and near-native configurations are recovered

**Figure 2:**
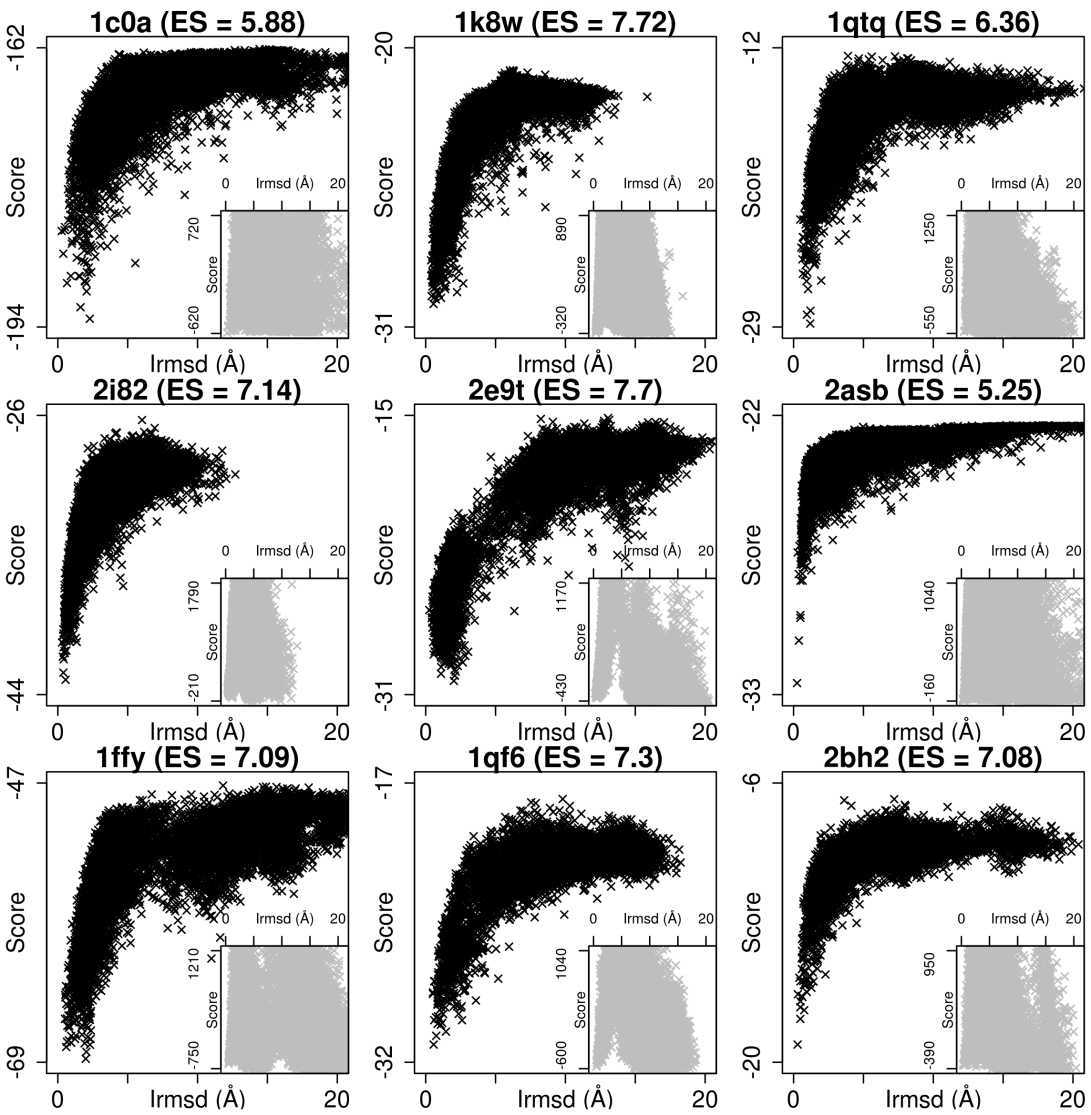
Energy vs Irmsd for 9 protein-RNA complexes. The 10,000 conformations evaluated for our optimized RosettaDock scoring function are shown in black. On each plot, the bottom left panel shows the equivalent non-optimized RosettaDock result.

A data based docking procedure for protein-RNA complexes should first be able to recover the native and close-to-native states for a reference set of complexes. This is assessed by a careful cross-validation setting. In this study we assessed the performance of our learning procedure by plotting Energy vs. Irmsd and checking the enrichment scores of our procedure relatively to the Rosetta CAPRI default. Figure 2 shows detailed results for nine different complexes (the remaining plots can be found in Figure S2). Interestingly, while only one complex (2e9t) shows a funnel in the default Rosetta version, none of the others do. Funnels can be found however on all the optimized scoring function plots that correlate to a high Enrichment Score. While not all complexes in the dataset display such a good conformation selection, the optimized scoring always performs better than the default RosettaDock setting and seems suitable for prediction.

**Figure 3:**
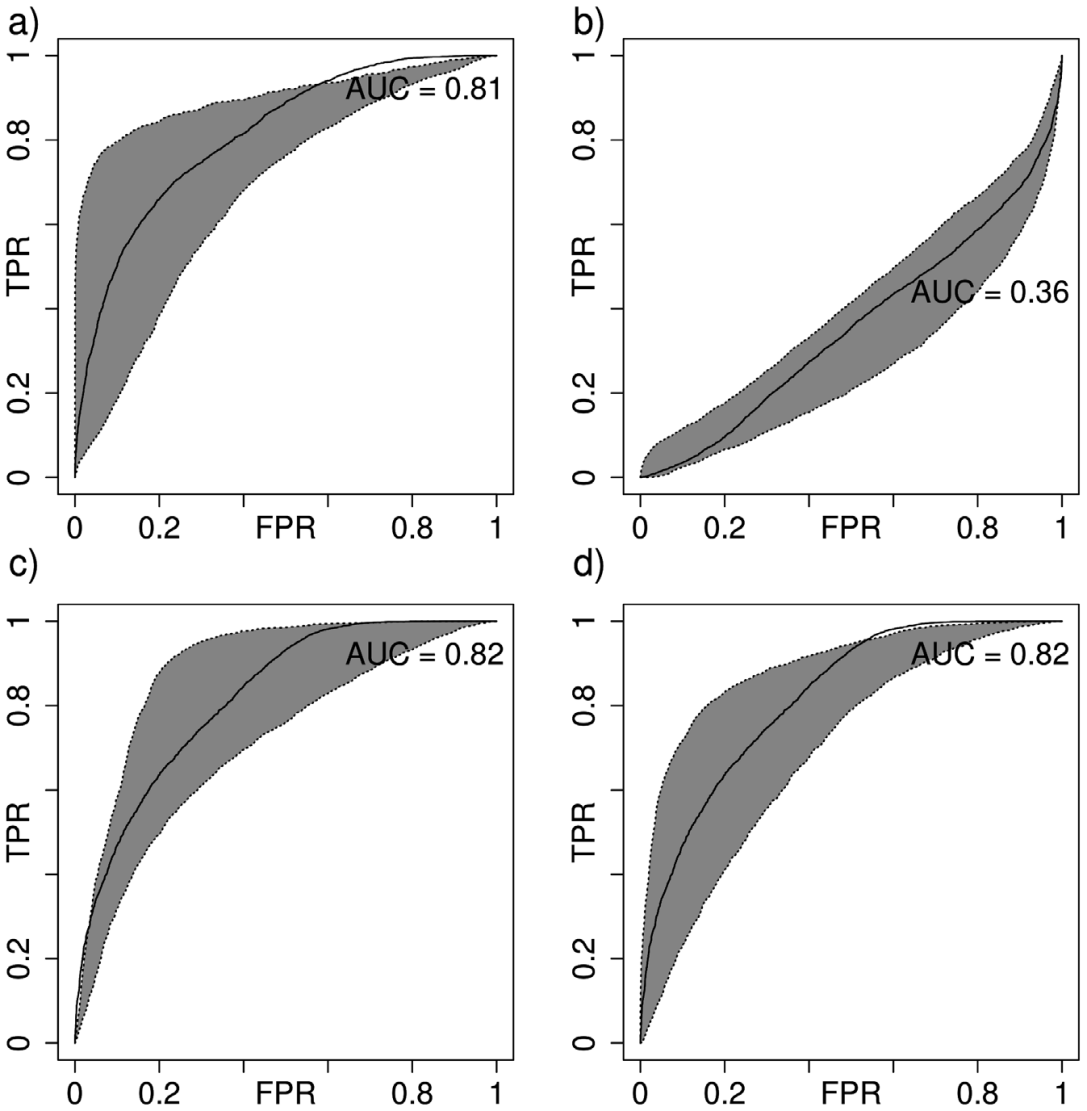
ROC Curves (True Positive Rate -TPR- vs. False Positive Rate -FPR-). (a) ROGER logistic scoring function, (b) Default RosettaDock score, (c) the whole protein-RNA benchmark I, (d) the whole protein-RNA benchmark II. The median ROC Area Under the Curve (AUC) is shown as a black line. The dotted lines delimiting the gray area correspond to the 1st and 3rd quartiles. Reported on the plots are ROC-AUC values for the median.

The machine learning procedure was also assessed separately by plotting the ROC curves in the leave-one-*pdb*-out setting. Figure 3 (panels a and b) shows the ROC curves for the optimized and default RosettaDock scoring functions respectively. While the default strategy does not show any discrimination power, our optimized function performs very well. In particular, at the origin, the ROC curve is very steep. This is especially interesting as in the CAPRI challenge only 10 putative conformations can be submitted and in any experimental setting, not more than 100 can be easily tested. Table 1 reports the statistics for the previously mentioned complexes and confirms that a large number of near-native conformations can be found in the top10 and top100 conformations, making the optimized score suitable for prediction (Results on the whole reference set are available in S3). The ROC-AUC often shows larger improvements than the Enrichment Scores as the near-native category for the AUC is defined by a 5 Å threshold (the ES uses the top10% which is generally different than 5 Å).

**Table 1:** Leave-one-pdb-out scoring statistics for nine protein-RNA complexes.

PDB code	Enrichment Score		Top10			Top100		# of near native	AUC	
	Default	Roger	Default	Roger	expected	Default	Roger		Default	Roger
1c0a	0.46	5.88	1	10	2.57	11	99	2568	30.41%	91.29%
1k8w	0.59	7.72	1	10	3.92	5	100	3916	35.28%	93.78%
1qtq	0.05	6.36	0	10	2.97	4	100	2971	30.37%	89.09%
2i82	3.45	7.14	6	10	6.91	60	100	6908	40.69%	84.12%
2e9t	0	7.7	0	10	1.69	0	100	1688	19.00%	99.80%
2asb	1.18	5.25	2	10	5.48	3	100	5475	45.69%	92.22%
1ffy	0.02	7.09	0	10	3.12	0	100	3121	42.32%	93.05%
1qf6	0.09	7.3	0	10	1.15	0	99	1154	23.09%	90.18%
2bh2	0.29	7.08	0	10	2.34	0	100	2340	32.26%	88.99%

Enrichment Score, 10 best energy candidates, 100 best energy candidates, number of near-native structures and Area Under the ROC Curve are reported for each native structure both using the non-optimized RosettaDock scoring function (Default) and our optimized scoring function (ROGER).

The strategy was then further evaluated on the Benchmark I and Benchmark II protein-RNA complex structures in a bound setting. Figure 3 (panels c and d) shows the ROC curves for both benchmarks. The ROC-AUC confirms that the optimized scoring function performs well in a prediction setting and is robust to the biological diversity and flexibility encountered in both benchmarks.

### Most of the best energy candidates are biologically relevant near-native candidates

**Figure 4:**
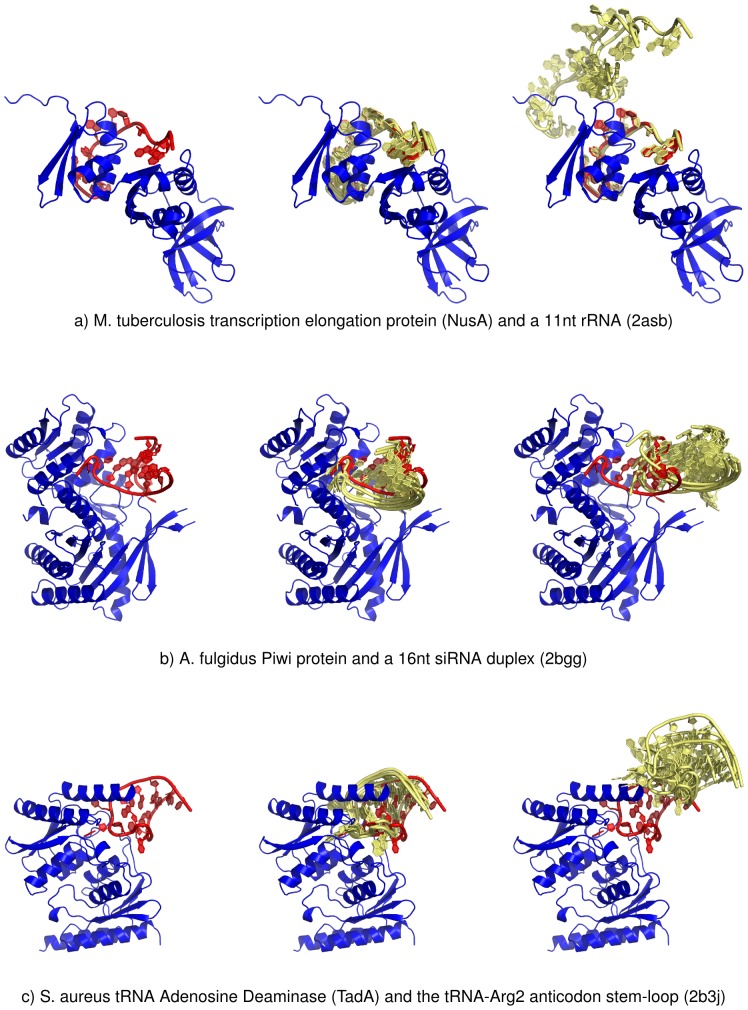
3D structures and predictions for three protein-RNA complexes (reference set). The protein is shown in blue, the native RNA in red and the RNA candidates in yellow. For each pdb example: (left) native structure, (middle) native structure superposed to the 5 best energy candidates from ROGER score and (right) native structure superposed to the 5 best energy decoys from RosettaDock default score.

The RosettaDock perturbation generation for the conformations ensures that the packing at the interface is relatively correct. Visual inspection shows that the conformations of best energy conformations are relevant from a biological perspective (interface area, contacts, clashes…). Figure 4 shows the 5 best energy candidates are very close to the native structure (bound setting). When various interface cavities are available for the docking (e.g. Figure 4b), the optimized function also clearly selects the right interface despite the atomic contacts being reasonable in both putative cavities. The default RosettaDock scoring function does select reasonably packed conformation but not always the right interface location.

### Optimized weights and interface H-bonding network

**Figure 5:**
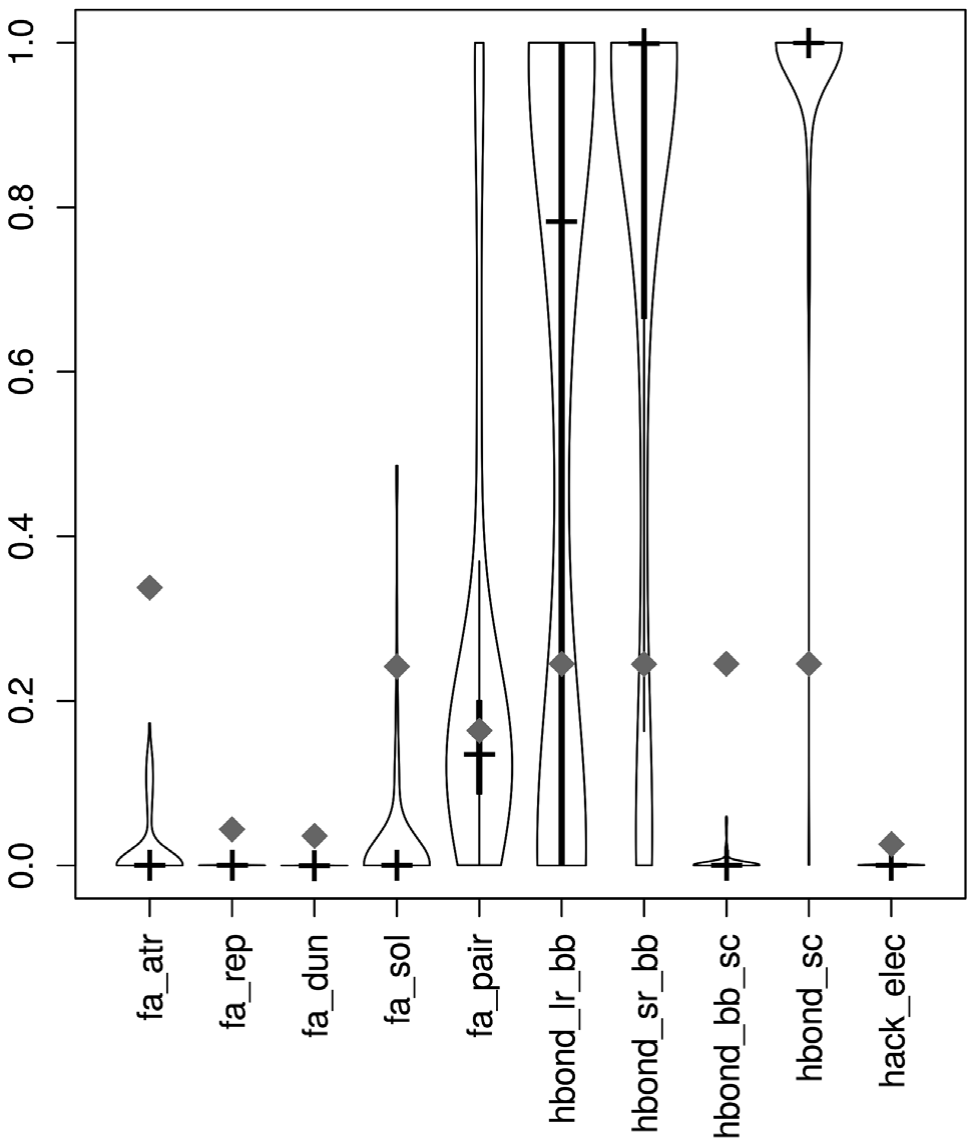
Violinplots of weights for the ROGER optimized RosettaDock scoring function. Default reference weights are shown in grey diamonds. fa_atr and fa_rep represent Lennard-Jones terms (attractive and repulsive). fa_dun corresponds to the internal energy of sidechain rotamers as derived from Dunbrack's; fa_sol is the Lazaridis Karplus solvation energy and fa_pair is the statistics based pair term, known to favour salt bridges for proteins. Remaining are H bond terms for long-range and short-range interactions for both backbone (bb) and side-chain (sc) terms. Last term (hack_elec) represents the empirical electrostatics contribution.

In a bound setting, for protein-RNA, the relative influence of the parameters shows that the H-bond network is extremely important and must be maintained. Figure 5 shows the weights obtained for the RosettaDock scoring function by optimization. H-bond terms involving the backbone are high at short range but also at long range. Unsurprisingly the H-bonding terms of the side chains are extremely important both for single and double strand RNAs (data not shown). Except for the pair term, most of the other terms have a very small influence. Other than the putative H-bonding network, only the pair terms have some importance. This is in accordance with the previous pair scoring functions developed for protein-RNA docking [28]. The relative importance of the weights however has to be assessed keeping in mind the values of the terms cannot really be normalized in the same range. The Lennard-Jones terms not having influence might be due to the fact that the system is set up on perturbation decoys generated by RosettaDock. By definition these will have a relatively good packing and clashing or too distant conformation will be left out without having to use the scoring function. To ensure the biophysical interpretation of the sign of the weights was compatible with our results, we also tried to optimize the scoring function by allowing the weights in the [−1;1] and in the [−1;0] intervals [54]. This led to much less stable learning procedures and worse results. We also checked whether the structural nature of the RNA molecules (single-, double-strand, tRNA…) made a difference but could not find any remarkable pattern. Score being high-resolution in a bound setting, the atomic contacts are more significant than the overall shape criteria.

### Benchmarking bound and unbound docking

The scoring function was then assessed in both bound and unbound (protein and RNA when available) settings. Perturbation runs were performed in a bound setting on the 40 complexes of the benchmarks not in the reference set. Only the 6 pdb files corresponding to median, 1^st^ and 3^rd^ quartile ROC performance were assessed in a full docking run unbound setting (for computational reasons). Results can be found in Table 2 and Table S4. As it was the case in a bound setting where results are consistent with the ones obtained on the reference set with cross-validation, the increase in performance for the unbound setting is also very clear. Results also show that AUC and enrichment score alone are not sufficient to evaluate the procedure and that the E vs. rmsd plots have to be checked as the rmsd distribution among the decoys can vary: while the enrichment score can be poor, the selection can be very good. The E vs. rmsd plots show very sharp funnels (Figures S3 and S4).These may contain two or three very sharp peaks corresponding to small changes in the residue rotamers and/or to the H-bonding network. All peaks do however correspond to native conformations in the CAPRI definition. For some case, the results stay poor: to improve these results flexibility of RNA should be taken into account so as to provide a wide range of small rmsd.

**Table 2:** Scoring results on the unbound test set.

PDB code	Category Prot/RNA	Enrichment		Top10			Top100		# near native	AUC	
		Default	Roger	Default	Roger	Expected	Default	Roger		Default	Roger
1m5o	U/B	0.43	4.75	0	4	4.79	3	66	4790	24.89%	79.37%
1qtq	U/U	0.00	6.09	0	10	2.798	0	98	2798	24.27%	90.99%
1wpu	U/B	1.60	3.15	9	10	8.723	71	100	8723	46.74%	70.37%
1yvp	U/B	0.93	0.20	6	10	9.362	61	100	9362	28.69%	80.89%
1zbh	U/U	0.88	0.00	5	10	9.834	82	100	9834	12.44%	96.07%
2ad9	U/B	0.00	6.06	0	0	0.001	0	0	1	1.65%	97.78%

Enrichment Score, 10 best energy candidates, 100 best energy candidates, number of near-native structures and Area Under the ROC Curve are reported for each native structure both using the non-optimized RosettaDock scoring function (Default) and our optimized scoring function (ROGER).

### Limits

A current limit of our approach is the way RNA flexibility is handled. Handling RNA flexibility for RNA during docking is a very difficult task [13]. Thus, aside from hydrogen atoms and protein rotamers, flexibility is not well taken into account. This can however be handled by geometric sampling [55]. For small RNA molecules this lack of flexibility handling is a limitation that cannot allow for good results despite a good high-resolution scoring function as it calls for a preliminary sampling experiment. Modelling electrostatics is also a major issue when modelling RNA molecules: solvent and ions are often found at the interface and are still hard to predict [56]. In our reference set, the interaction between the mRNA binding domain of elongation factor SelB from E.coli in complex with SECIS RNA (PDB code 2pjp) is an example where the interface is mediated by sodium ions that our model does not take into account and for which we obtained very poor results (See Figure S5). While our approach could totally be adapted and used for protein-DNA complex prediction, providing the parameters are optimized on a suitable dataset, a similar effect where ions mediate the interaction would be seen. It is also unclear whether the changes and motifs occurring in the DNA double helix for binding could be well captured by this approach. In addition to limited flexibility treatment, this limits the current data based approaches.

## Conclusions

Protein-RNA complexes are undoubtedly a real challenge for the design of good docking scoring functions. Using a well curated dataset and a well-designed optimization strategy, we show that we could set up of an efficient protein-docking scoring function that can be used in RosettaDock and that can perform better than the existing option in both bound and unbound settings. While scoring can be improved, the nature of RNA makes the prediction experiment still difficult. Electrostatics plays a large role in RNA interactions and ions have to be modelled. Like ours, the data based approaches are limited by the relatively small number of structures available to take ions into account carefully. RNA flexibility modelling for docking is then the next challenge: while some strategies allow for conformation sampling, selection of one or several putative bound states for large cross-docking experiments are still out of reach for both modelling and computational reasons.

### Availability

The source code and files needed to modify RosettaDock 3.4 are available at: http://albios.saclay.inria.fr/rosettadockrna

## Supporting Information

Figure S1 **Model of a nucleic acid (uracile).** The phosphate group and the sugar heavy atoms are depicted in gray: (a) coarse-grained level with the centroid atom in red and (b) full-atom level with the base atoms in blue. The centroid is the geometric center of the heavy atoms.
(TIFF)

Figure S2 **Energy vs Irmsd for the whole reference dataset in a leave-one-pdb-out setting.**
(PDF)

Figure S3 **Energy vs Irmsd for the benchmark set in a bound setting.** The 10,000 conformations evaluated for our optimized Rosetta scoring function are shown in black. On each plot, the bottom left panel shows the equivalent non-optimized Rosetta result.
(PDF)

Figure S4 **Energy vs Irmsd for the unbound test set.** The 10,000 conformations evaluated for our optimized Rosetta scoring function are shown in black. On each plot, the bottom left panel show the equivalent non-optimized Rosetta result.
(PDF)

Figure S5 **Structure of the mRNA binding domain of elongation factor SelB from** ***E.coli*** **in complex with SECIS RNA (PDB code 2pjp).** Mg2+ ions (shown in yellow) are located at the interface and mediate the interaction.
(TIFF)

Table S1 **Protein-RNA complexes reference set from the PRIDB.** The rightmost column indicates putative redundancy with the docking benchmarks. The *Type* column refers to the structural family of the RNA molecule: single strand RNA (ssRNA), double strand RNA or single-stranded RNA of helical/paired structure (dsRNA) or transfer RNA (tRNA).
(PDF)

Table S2 **Protein-RNA complexes for the test set.** For each complex the unbound column for protein and RNA reports the PDB code of the unbound structures when available. The difficulty codes are taken from [36], [38].
(PDF)

Table S3 **Leave-one-pdb-out scoring statistics for the reference dataset.** Enrichment Score, 10 best energy candidates, 100 best energy candidates, number of near-native structures and Area Under the ROC Curve are reported for each native structure both using the non-optimized RosettaDock scoring function (Default) and our optimized scoring function (ROGER).
(PDF)

Table S4 **Scoring results on the bound benchmark test set.** Enrichment Score, 10 best energy candidates, 100 best energy candidates, number of near-native structures and Area Under the ROC Curve are reported for each native structure both using the non-optimized RosettaDock scoring function (Default) and our optimized scoring function (ROGER).
(PDF)
