# Supplementary figures and images for: Protein-RNA Complexes and Efficient Automatic Docking: Expanding RosettaDock Possibilities
Source: PLoS One. 2014 Sep 30;9(9):e108928. doi: 10.1371/journal.pone.0108928 (PMC4182525; doi:10.1371/journal.pone.0108928)

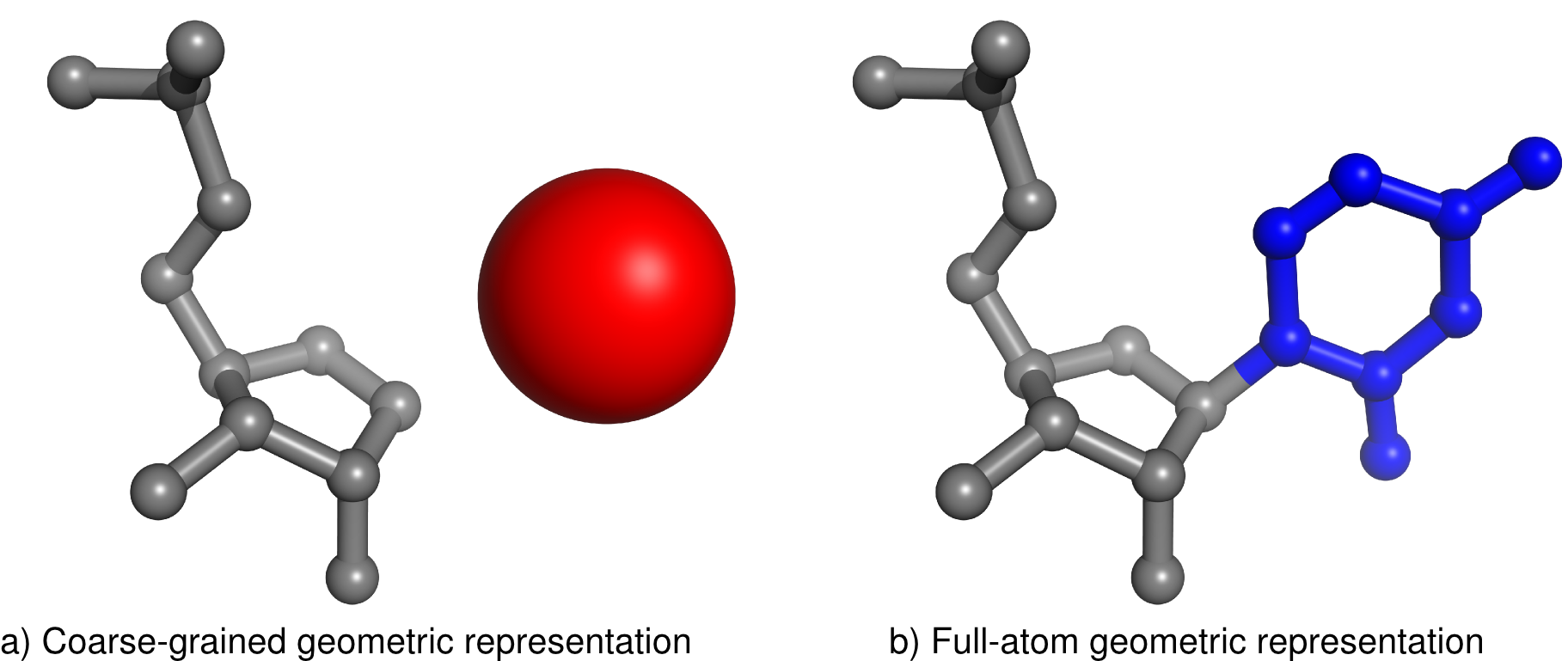

Supplement: Figure S1 — Model of a nucleic acid (uracile). The phosphate group and the sugar heavy atoms are depicted in gray: (a) coarse-grained level with the centroid atom in red and (b) full-atom level with the base atoms in blue. The centroid is the geometric center of the heavy atoms. (TIFF) [file pone.0108928.s001.tiff]

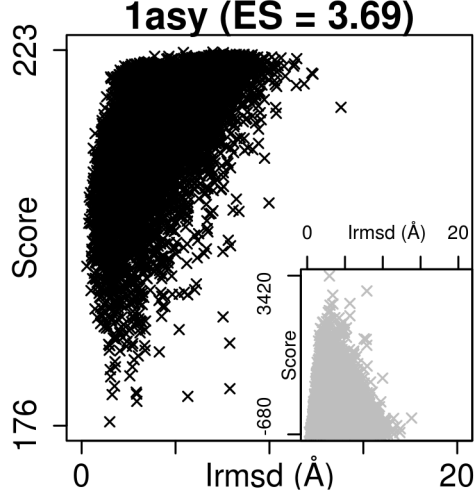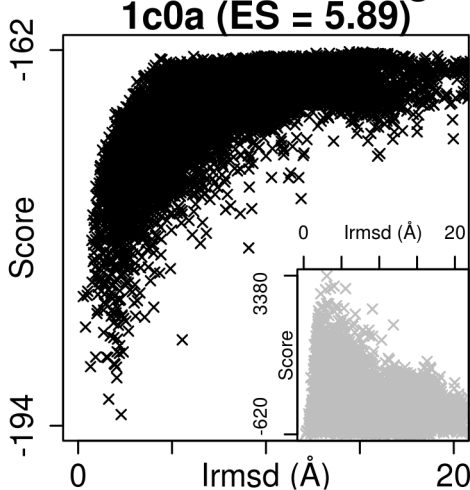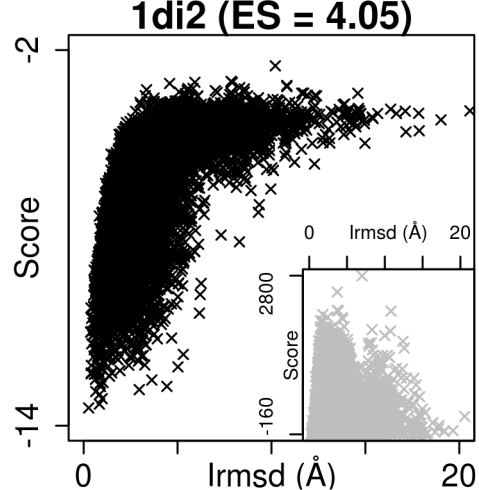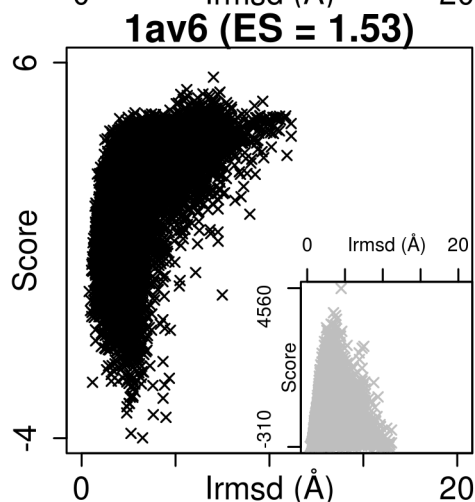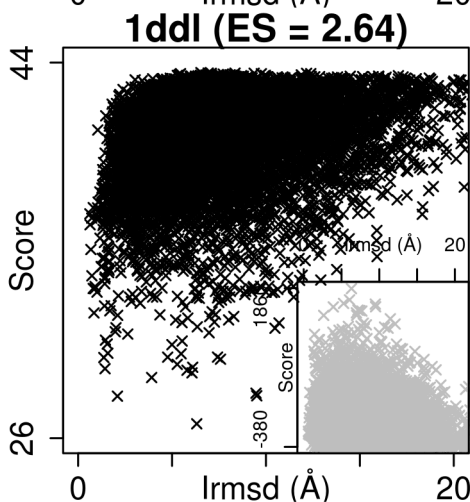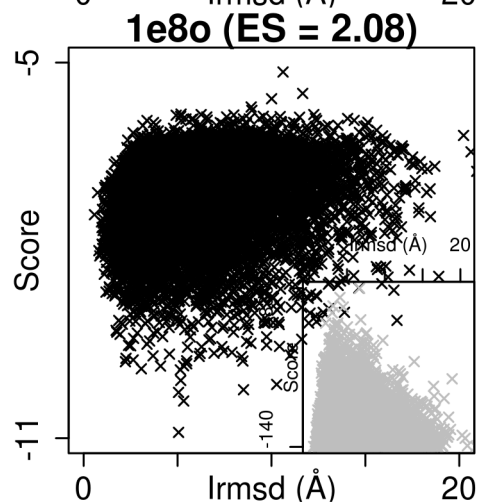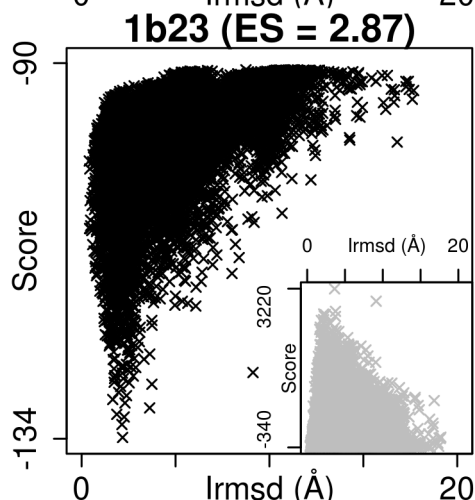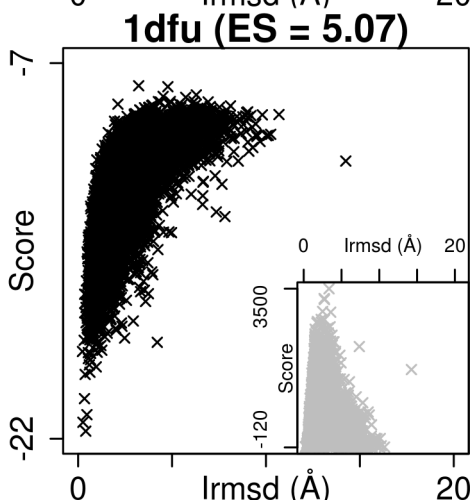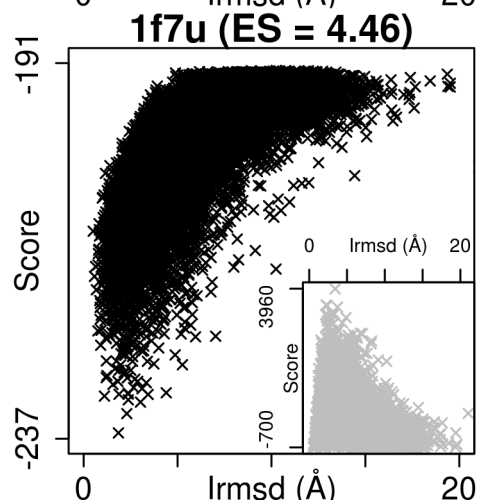

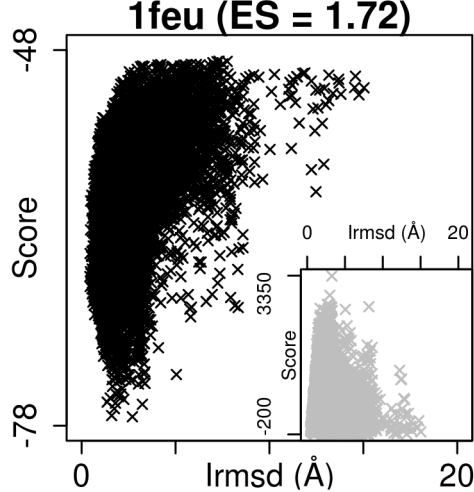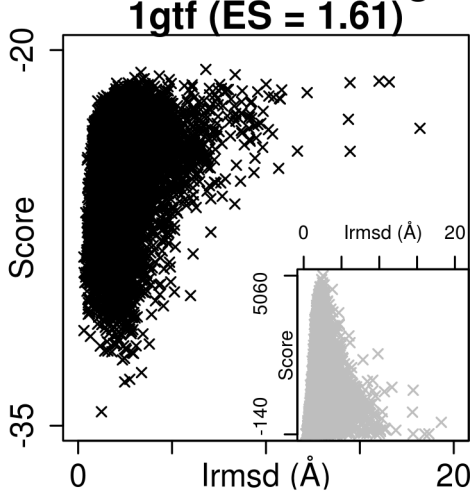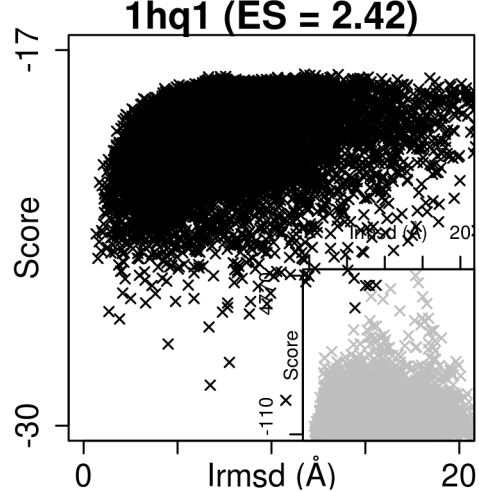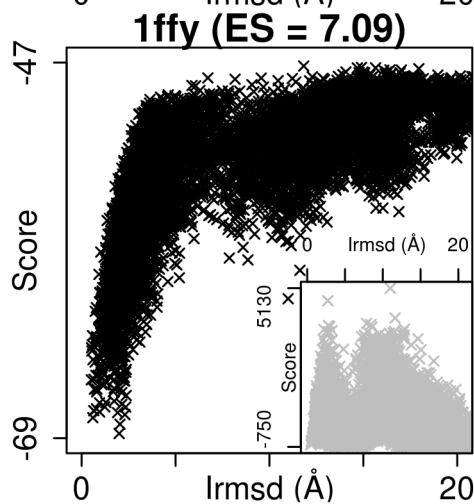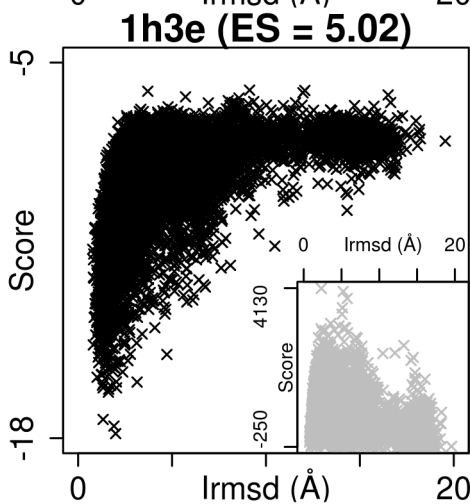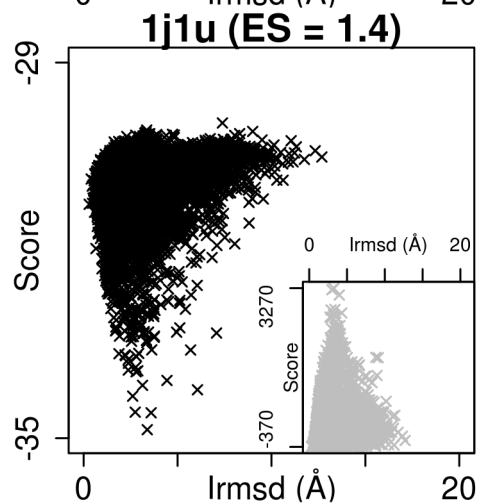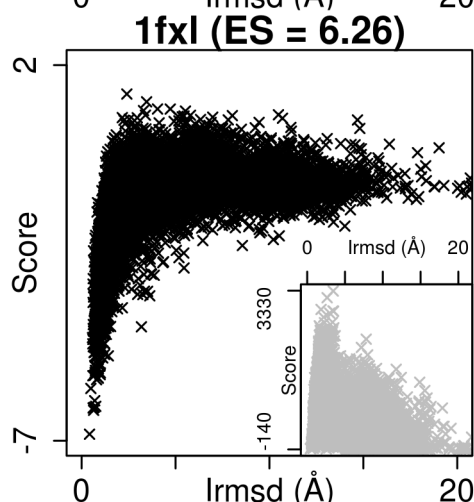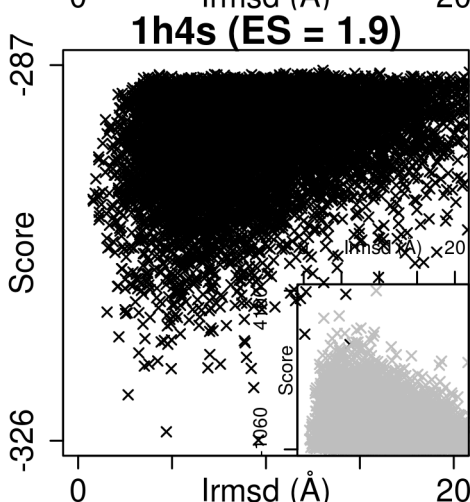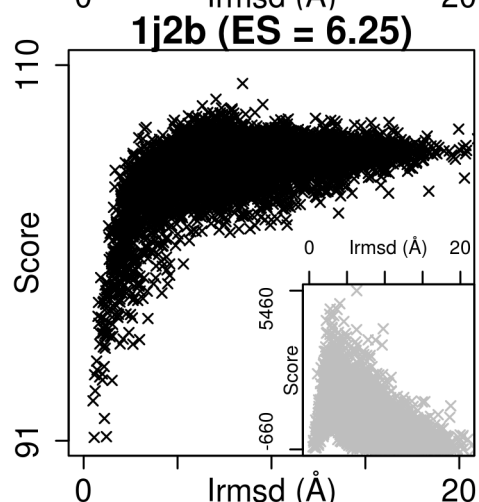

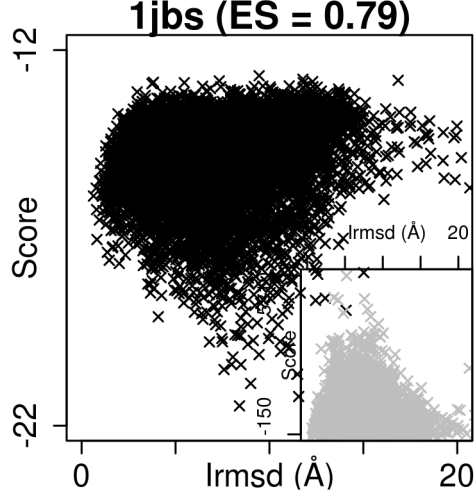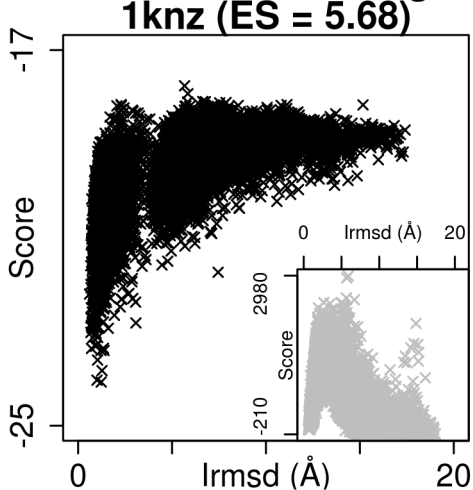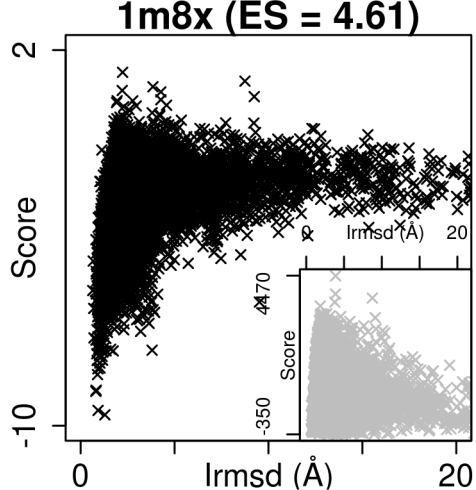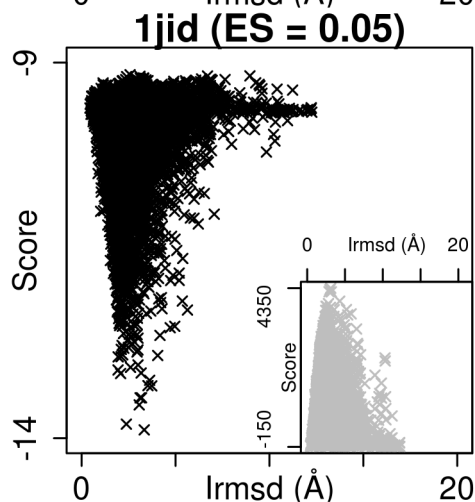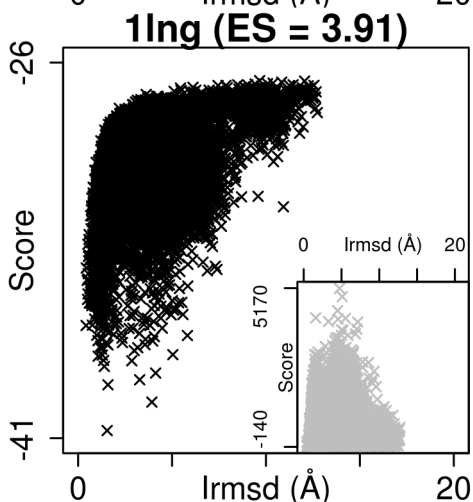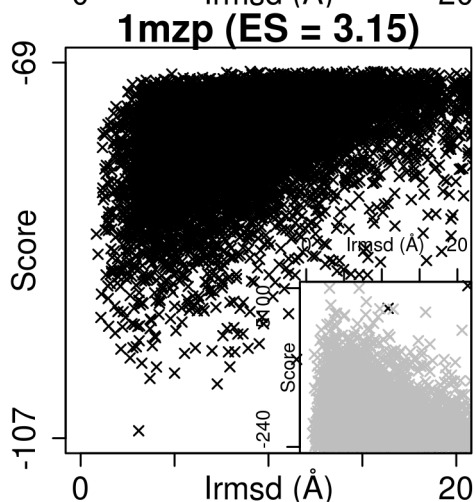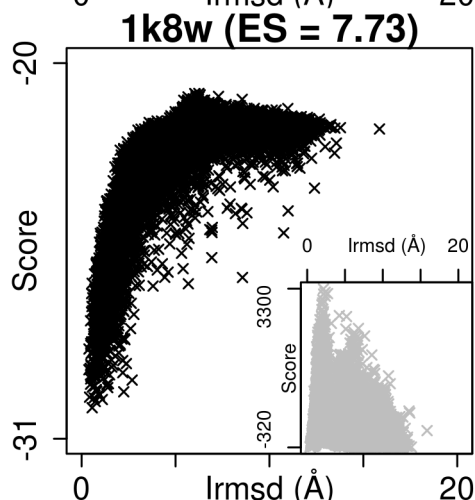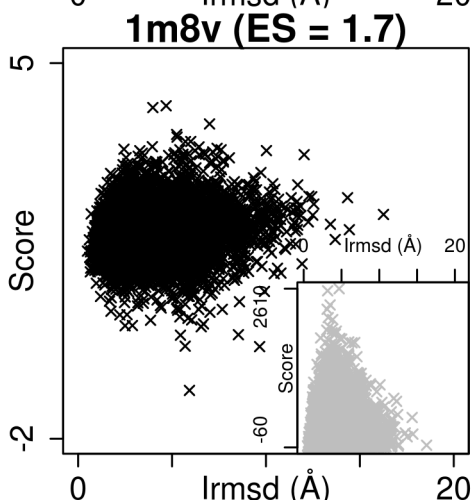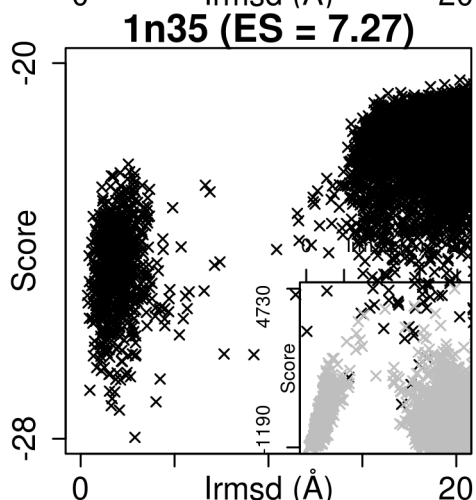

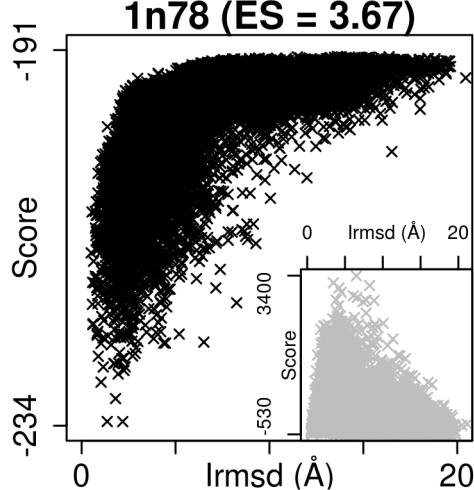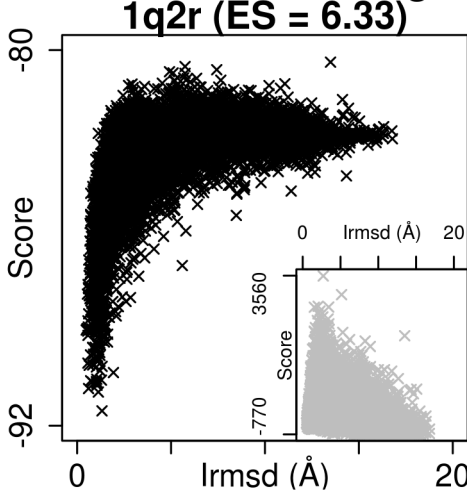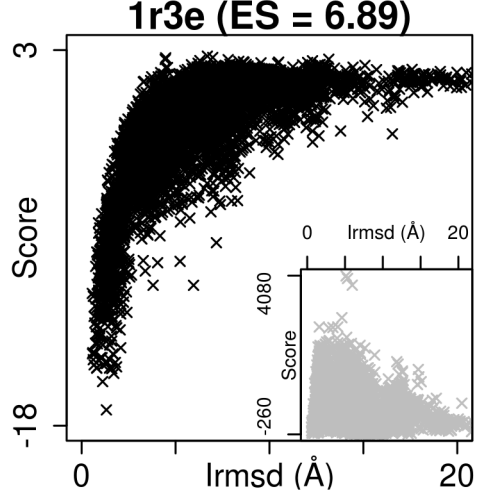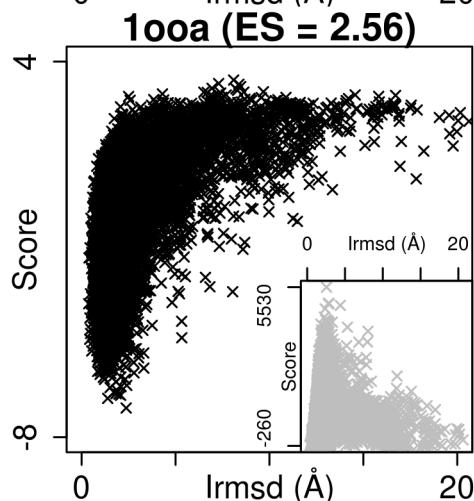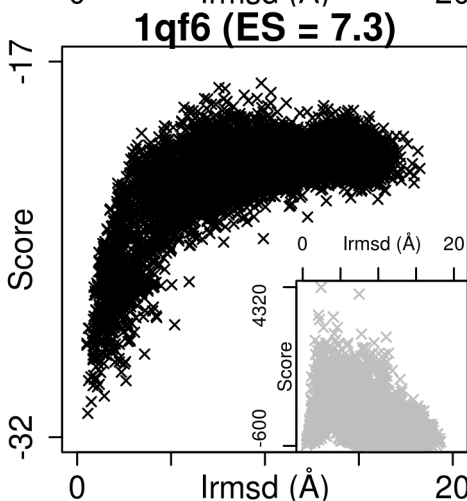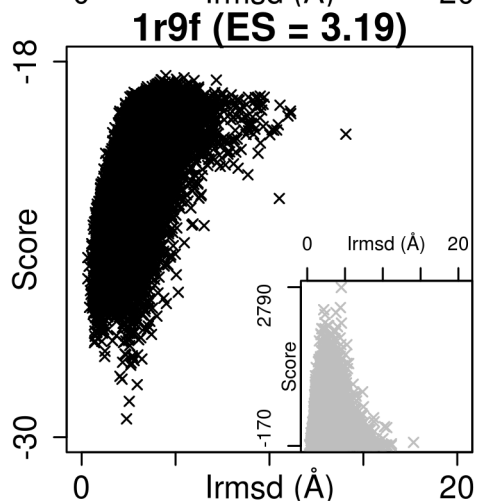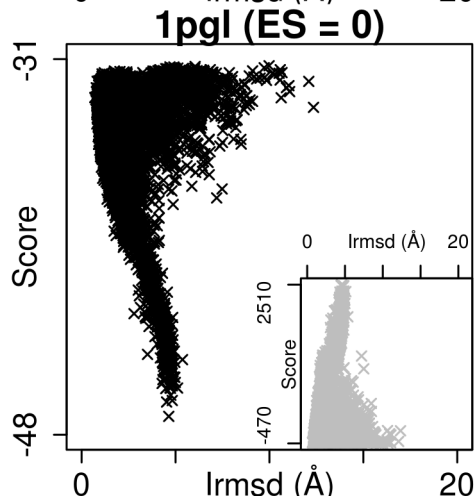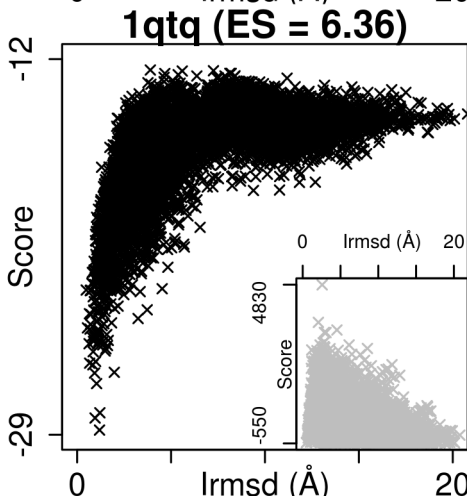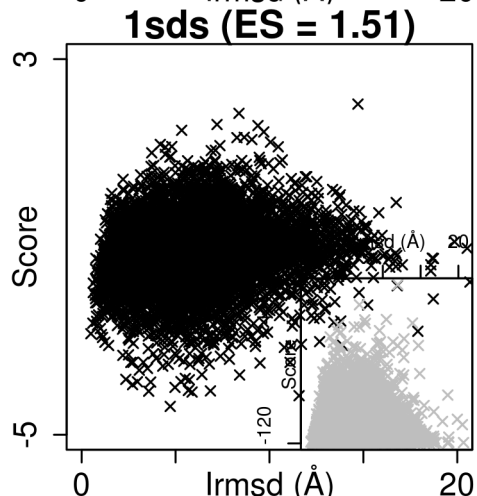

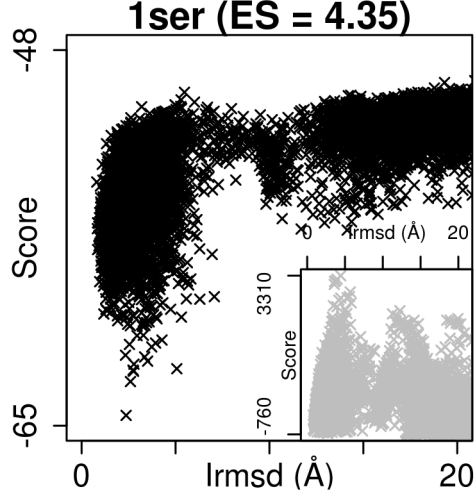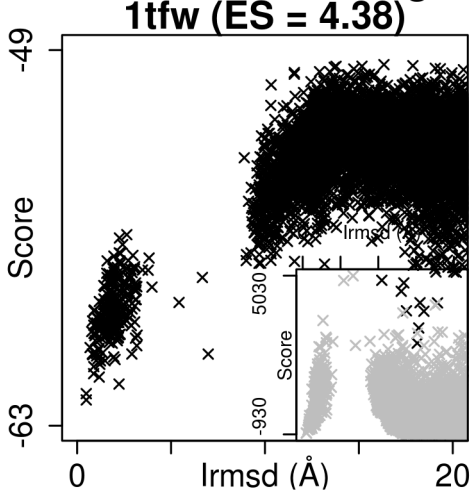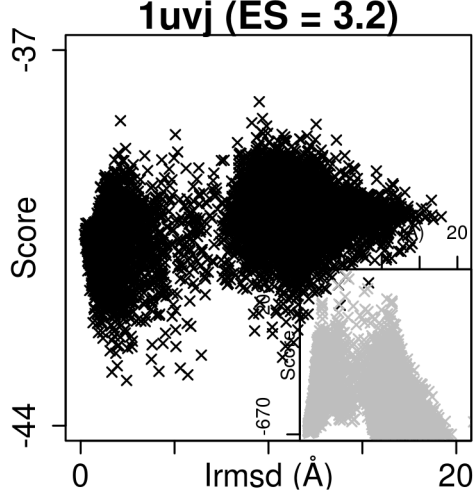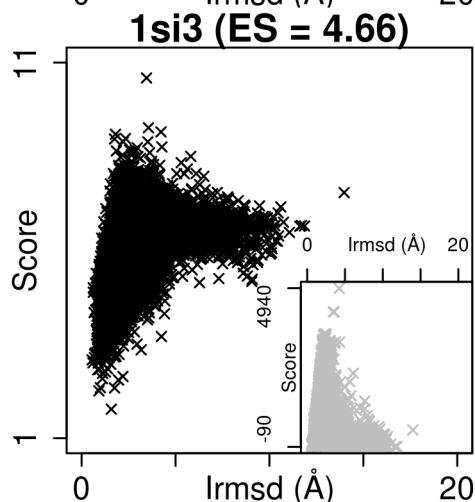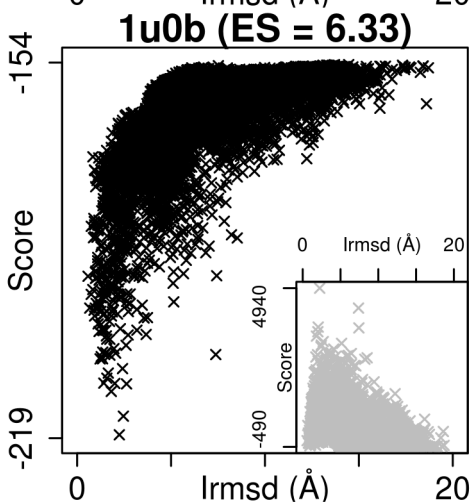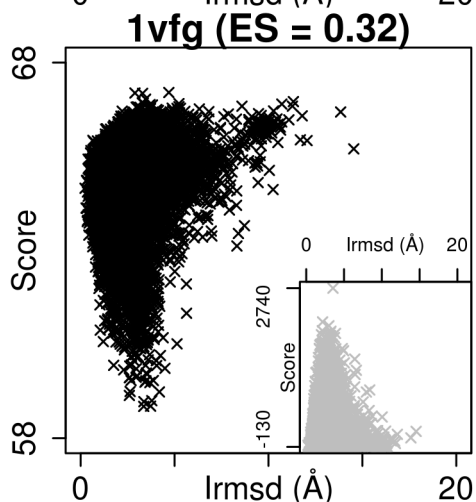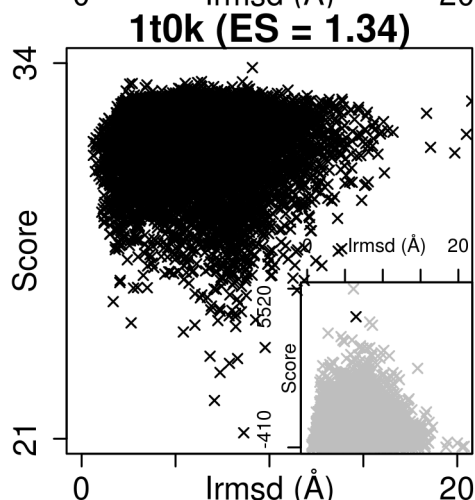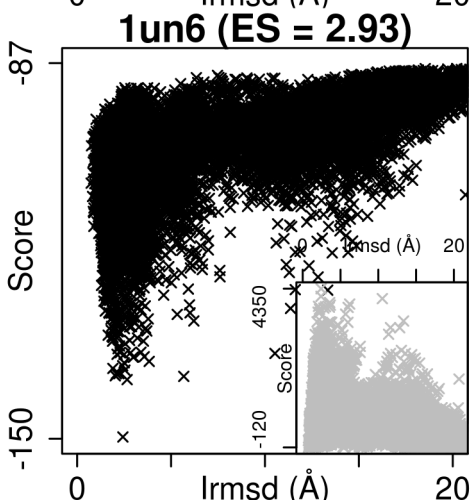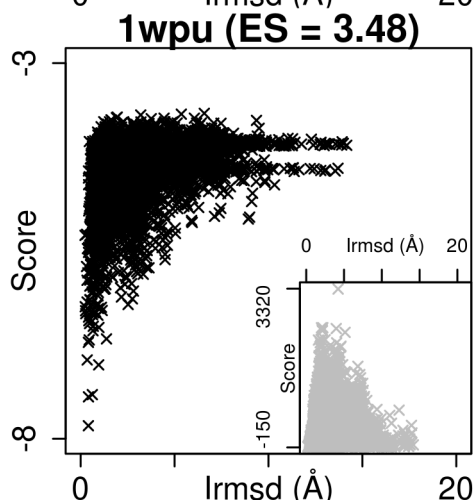

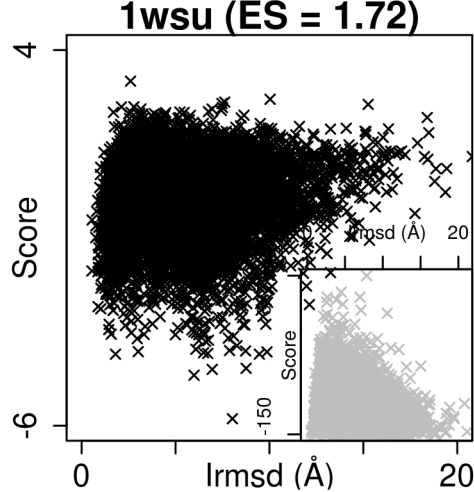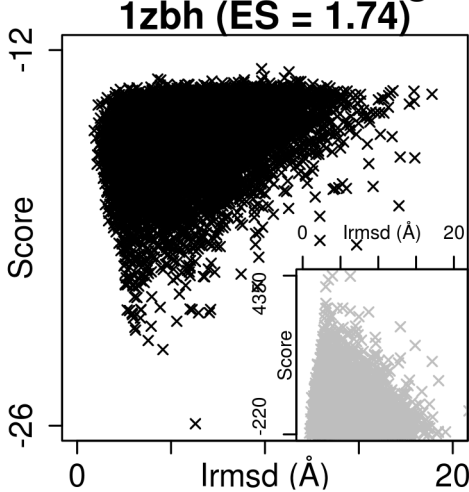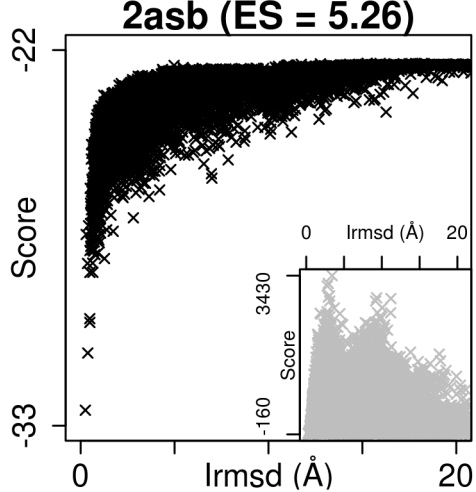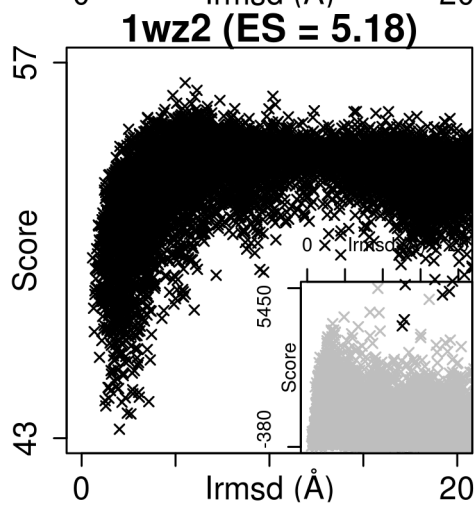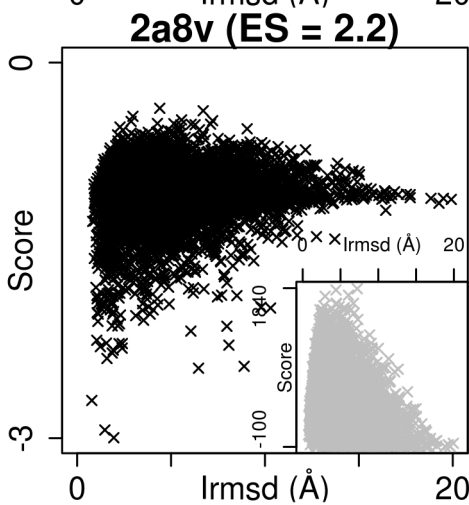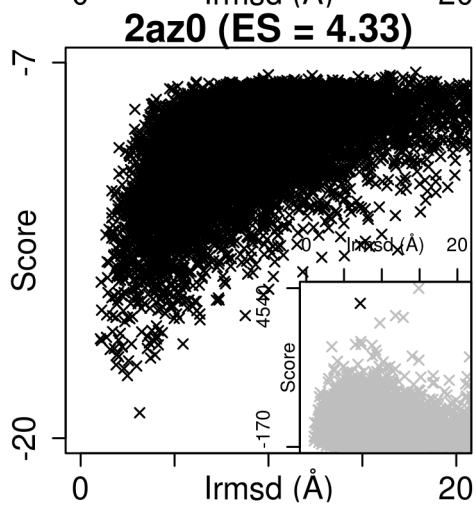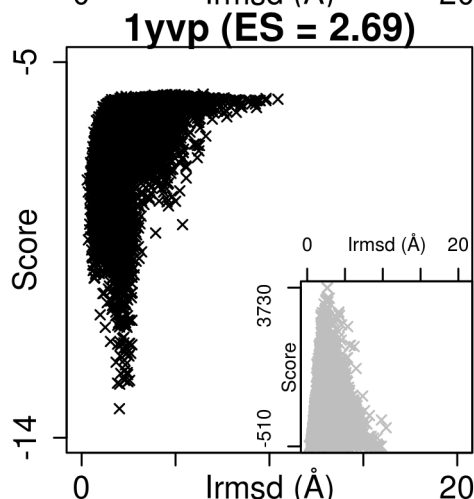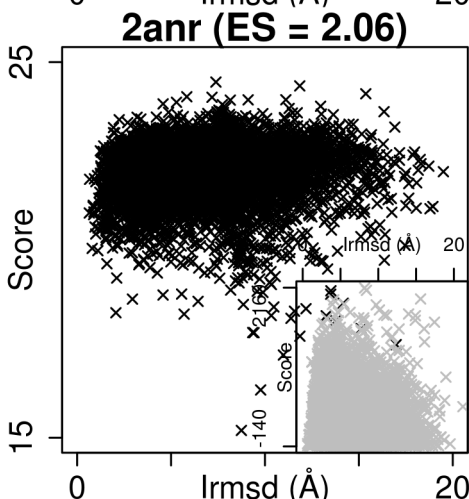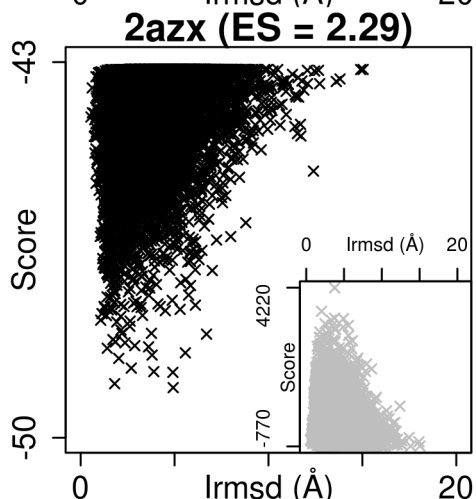

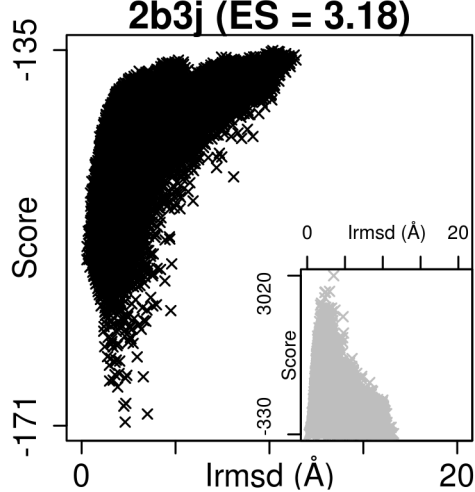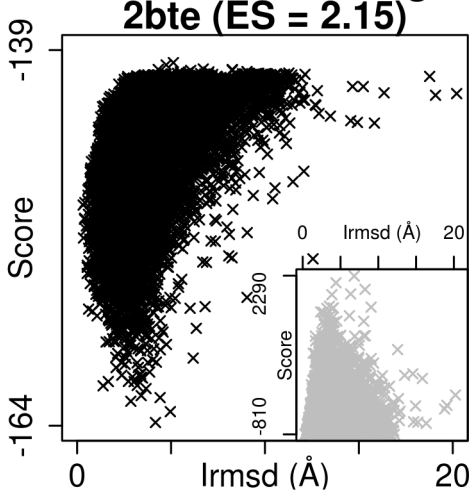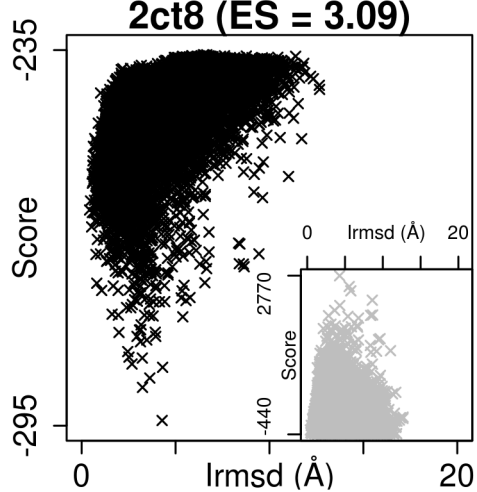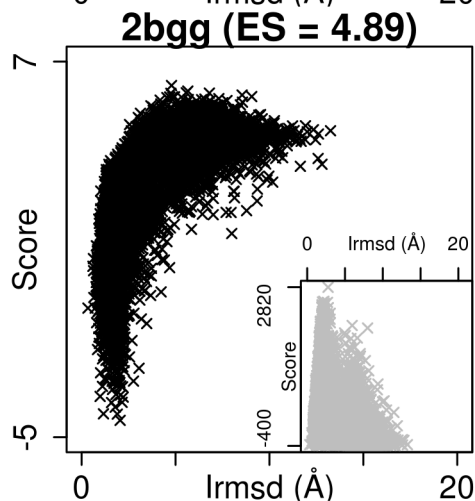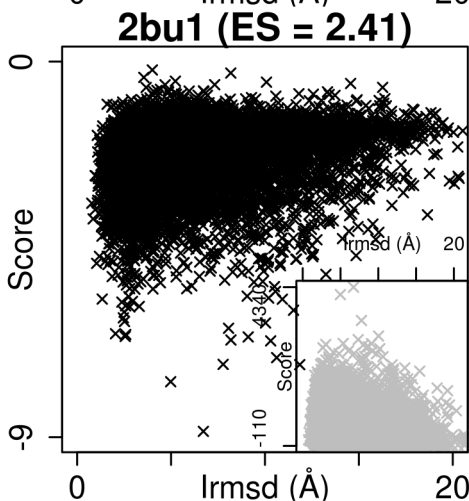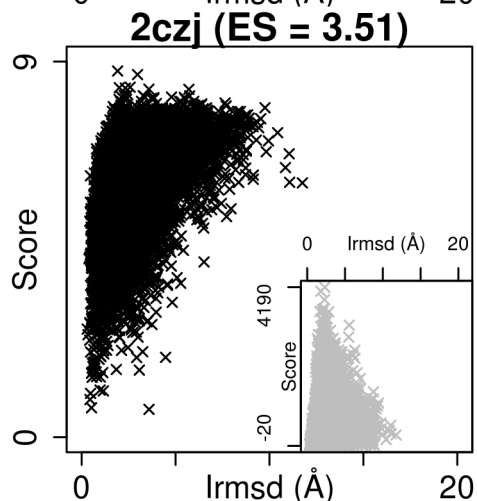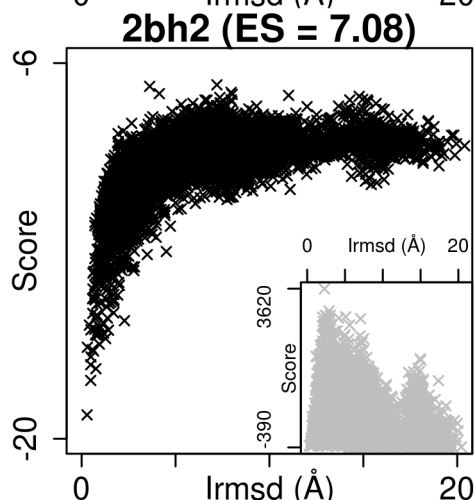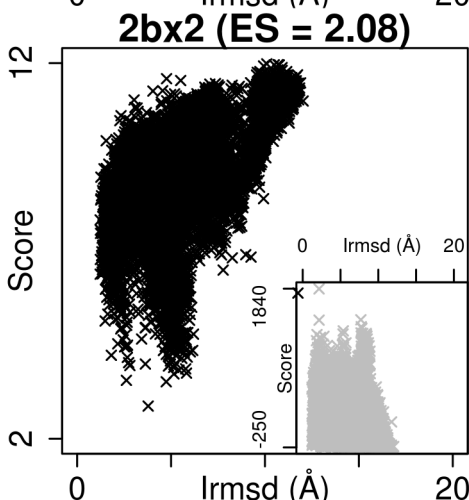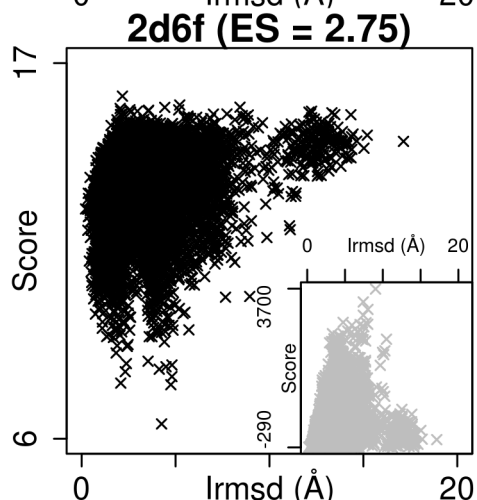

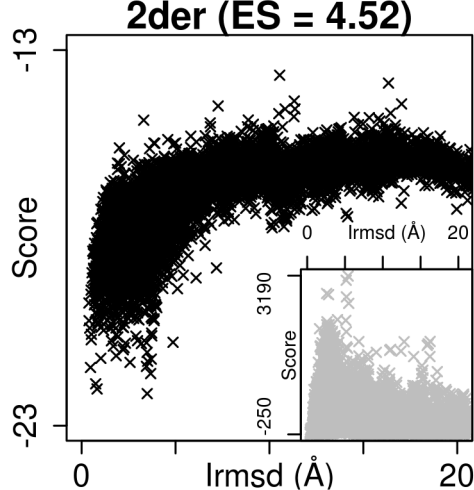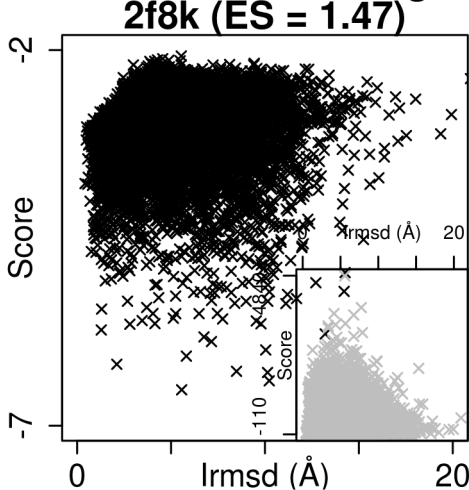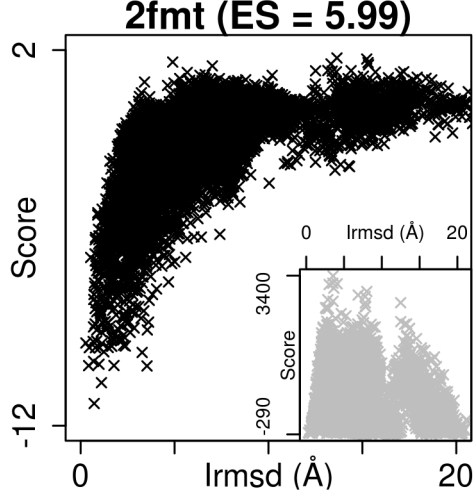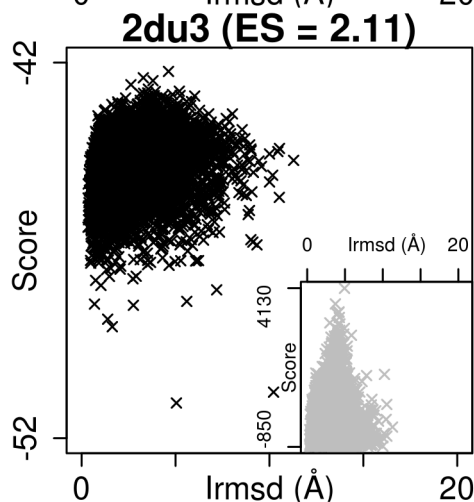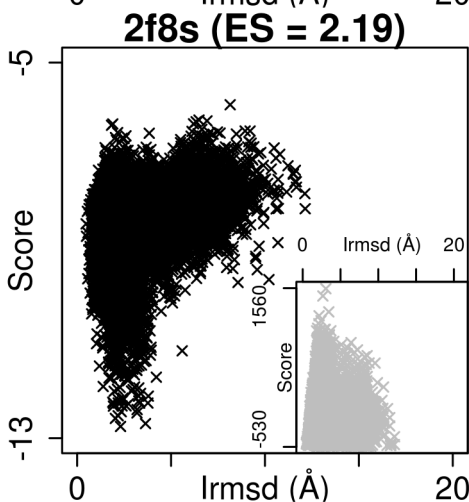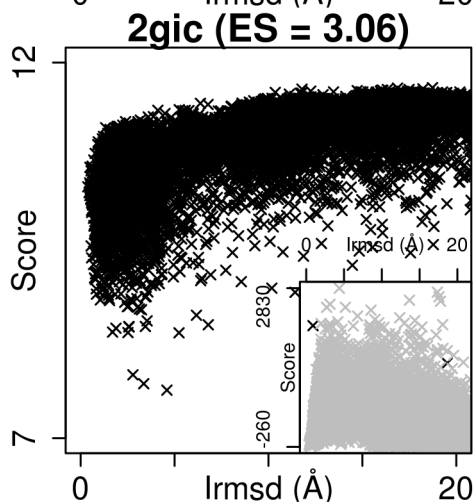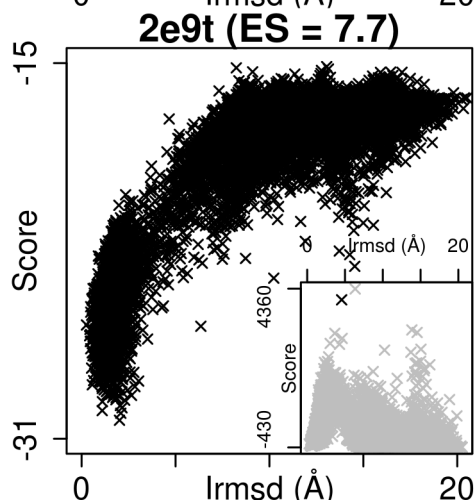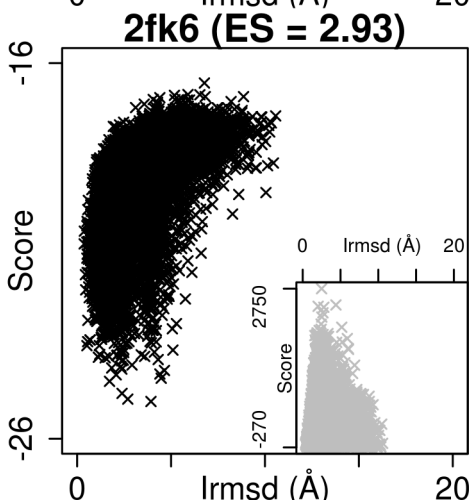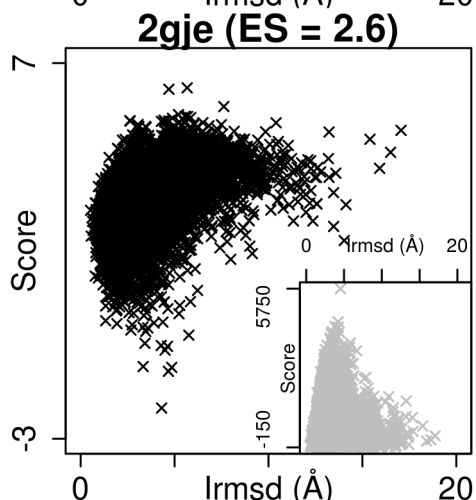

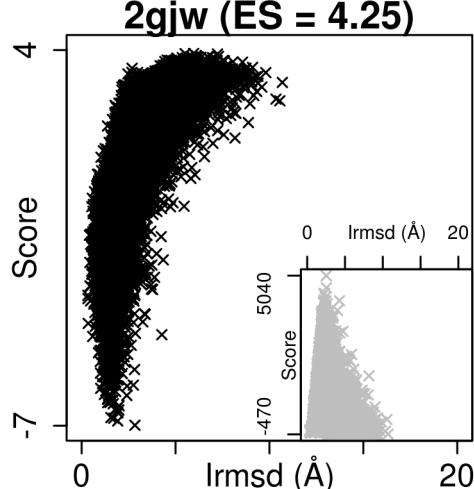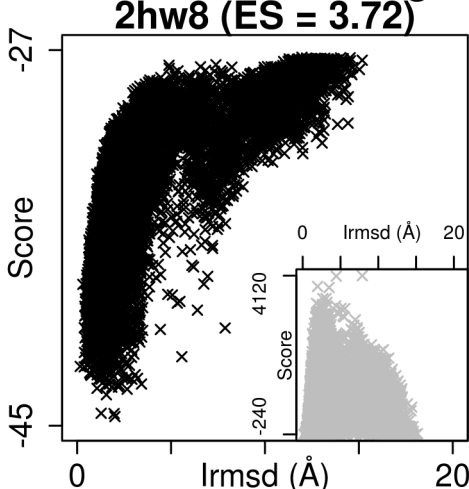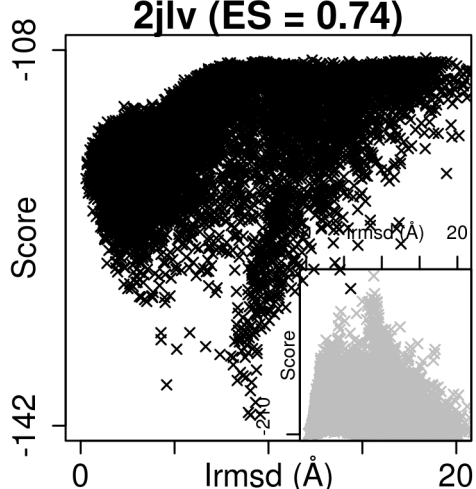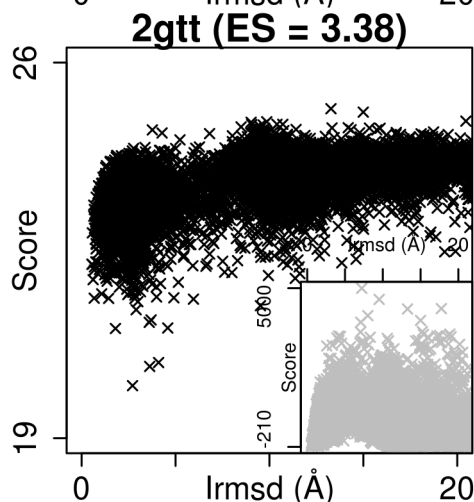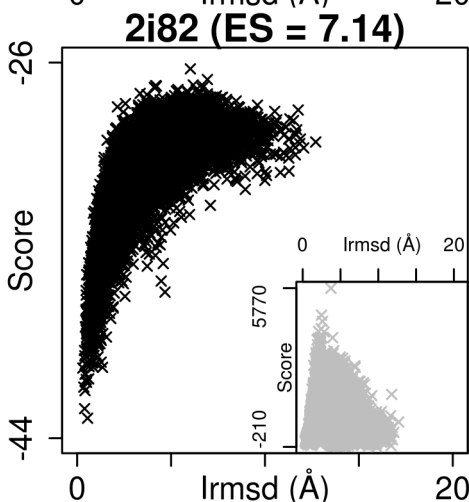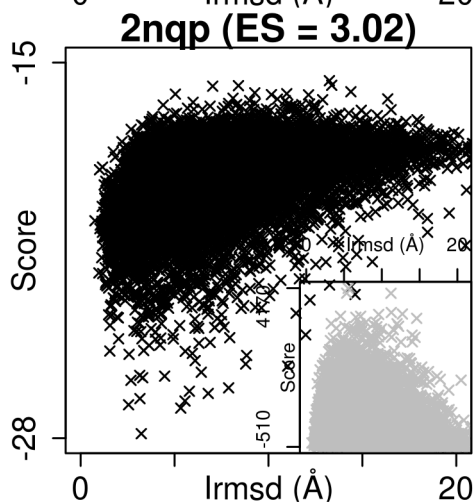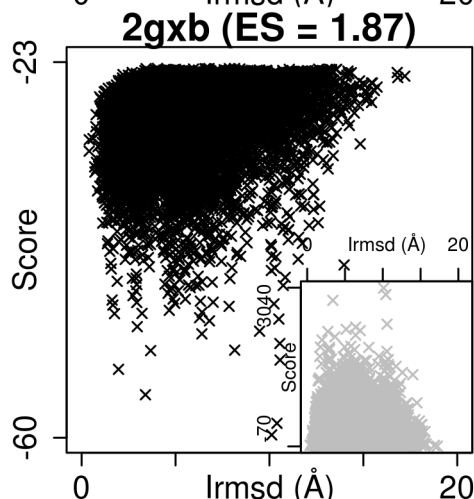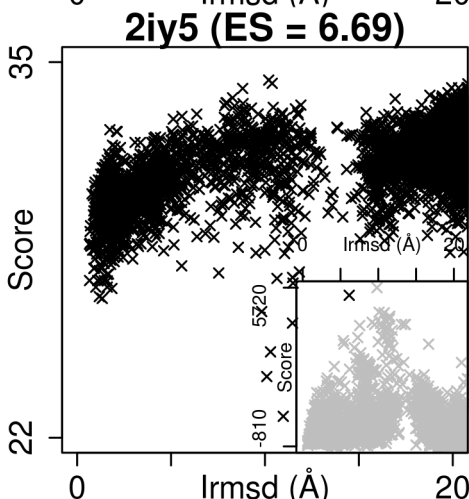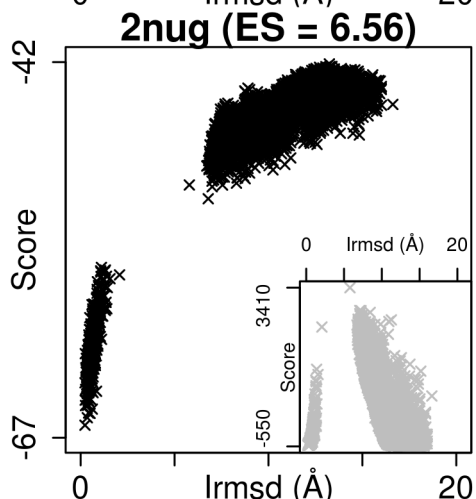

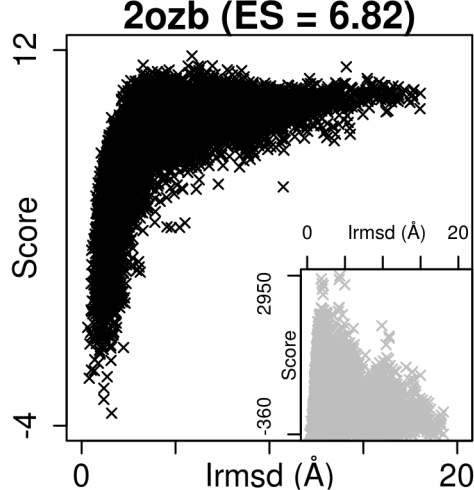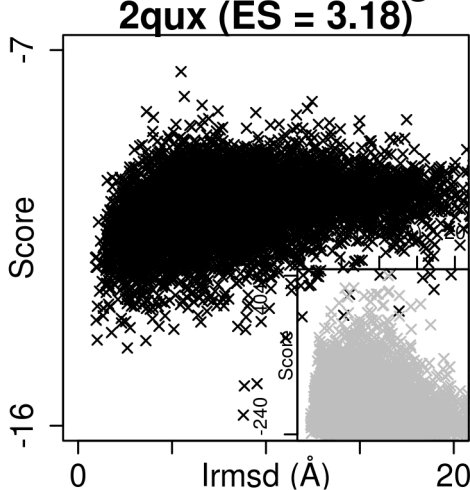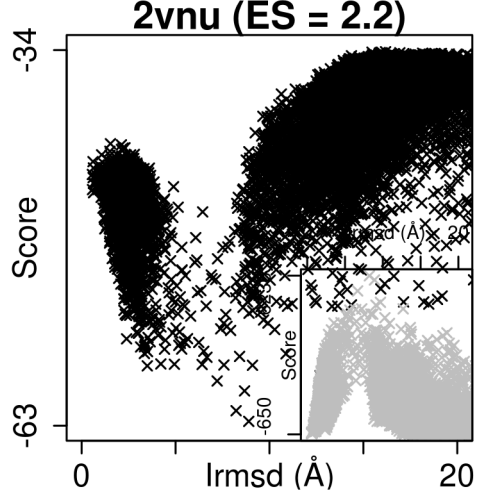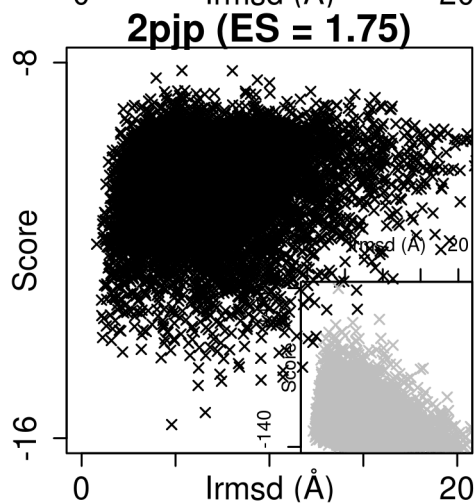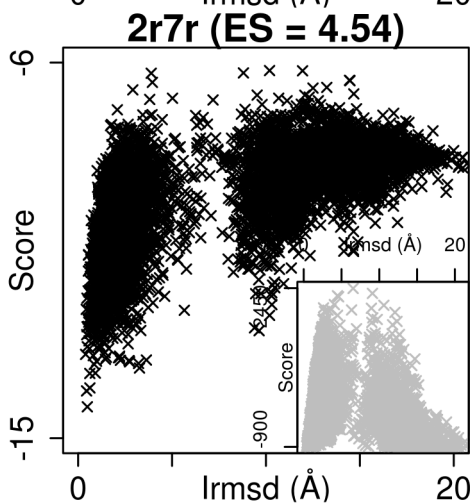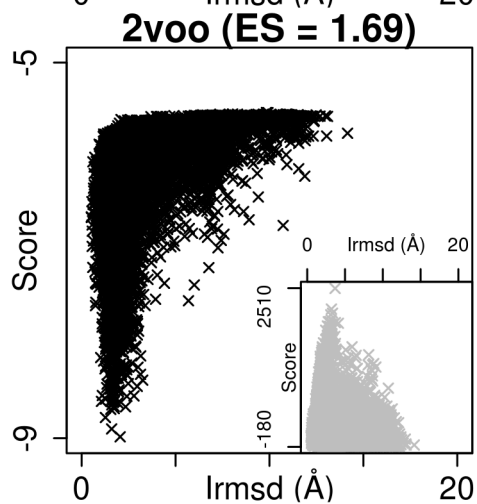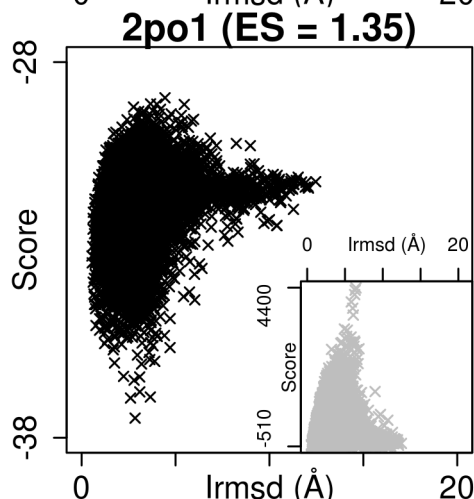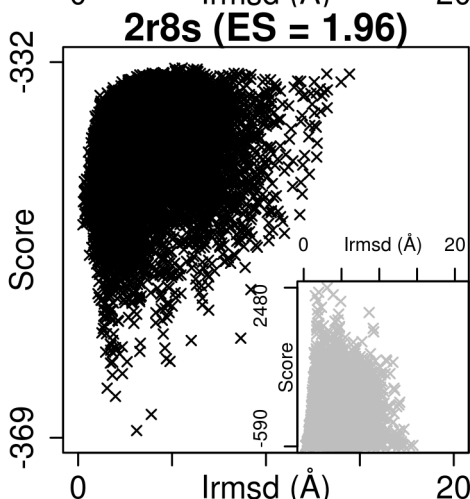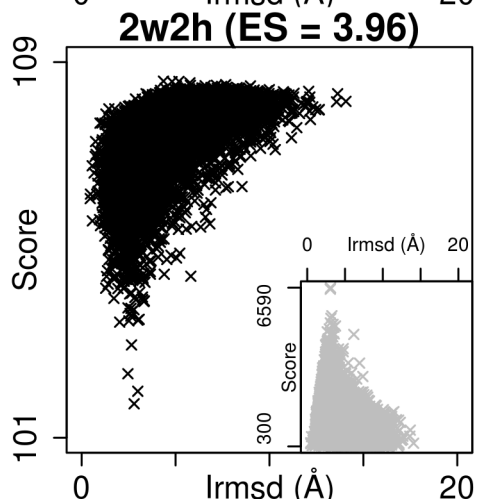

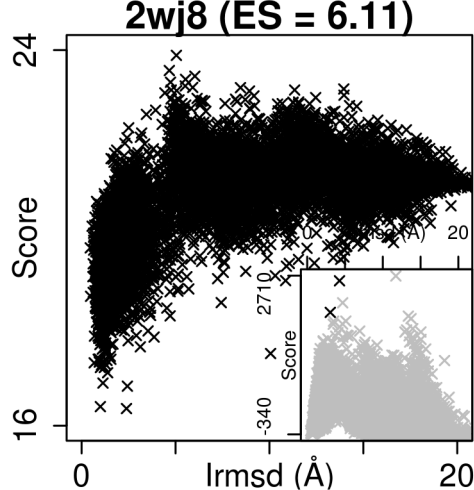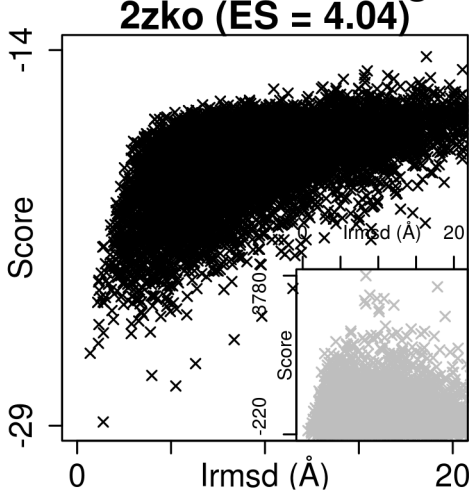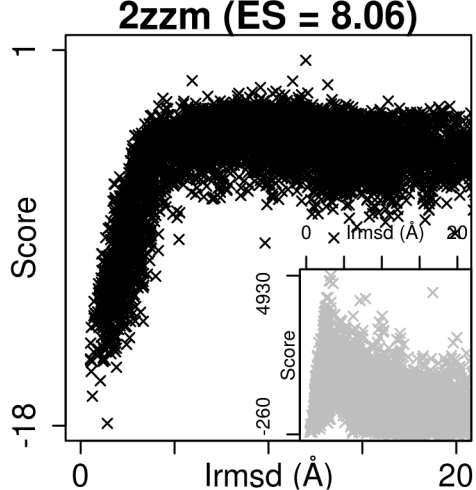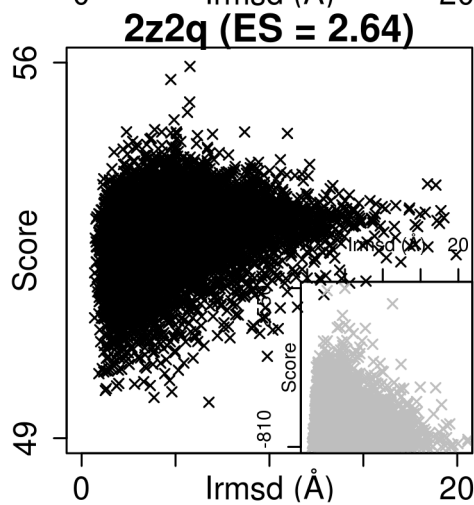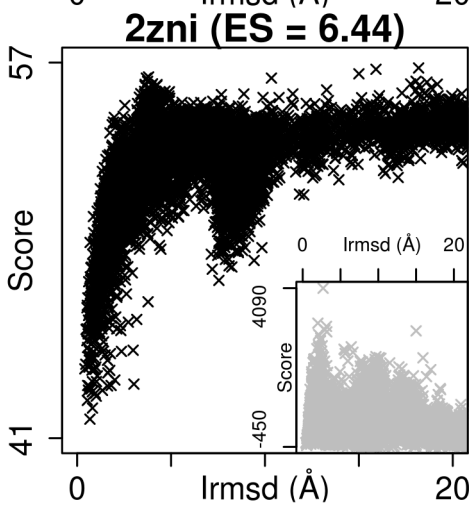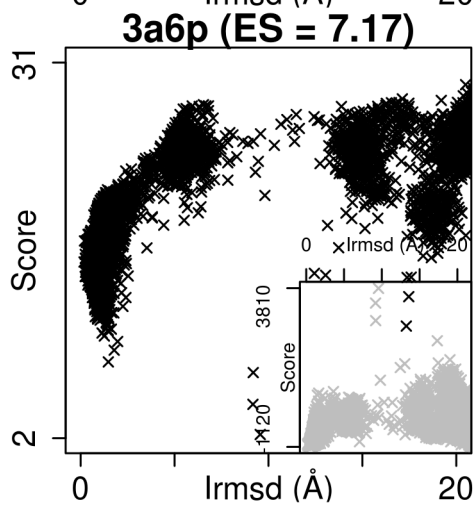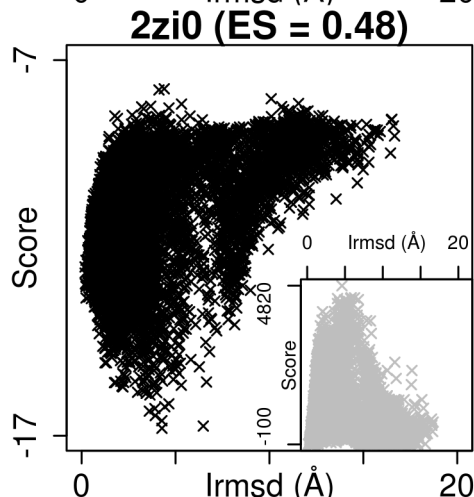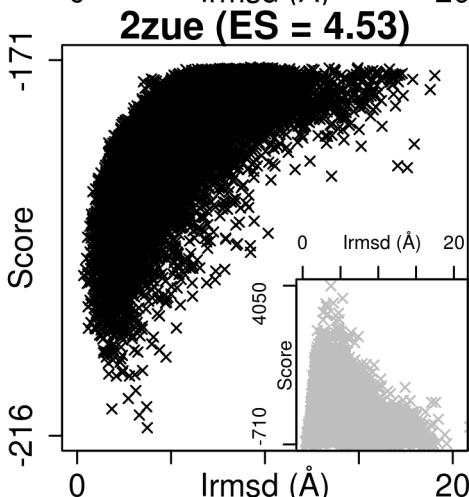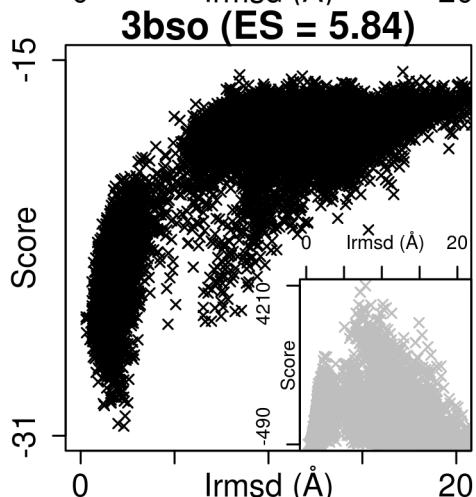

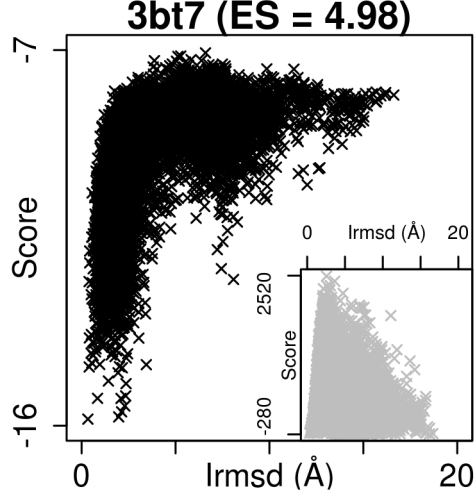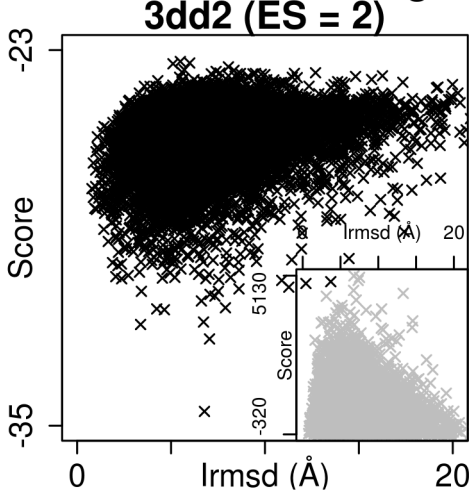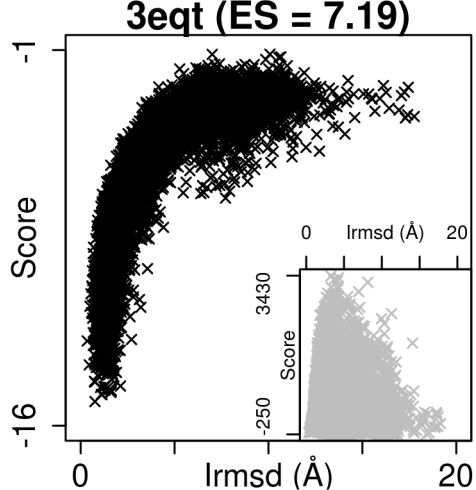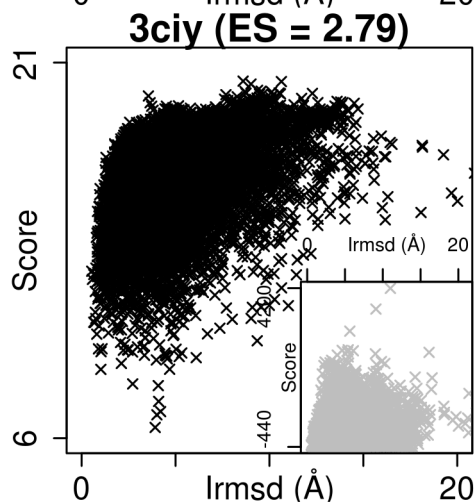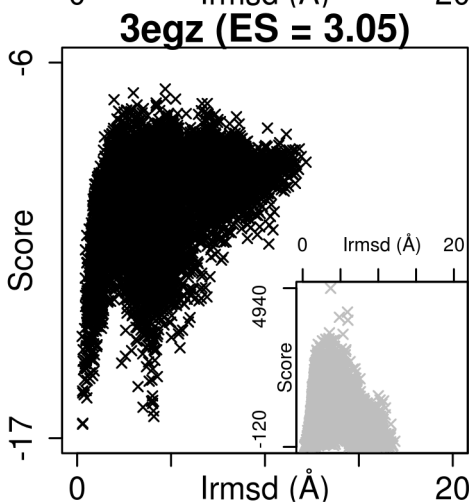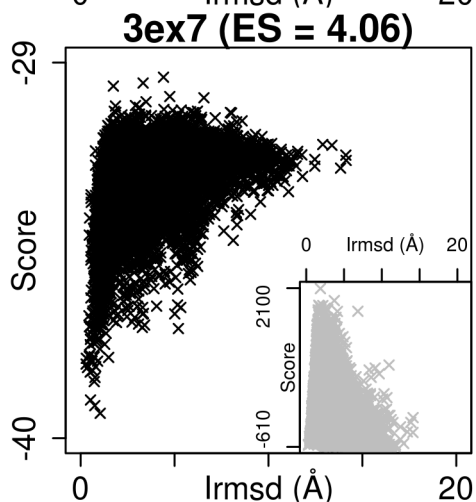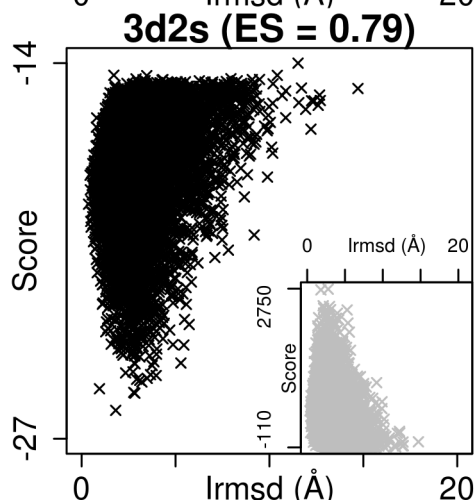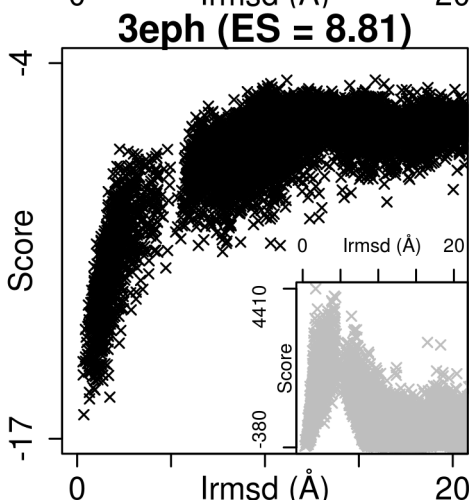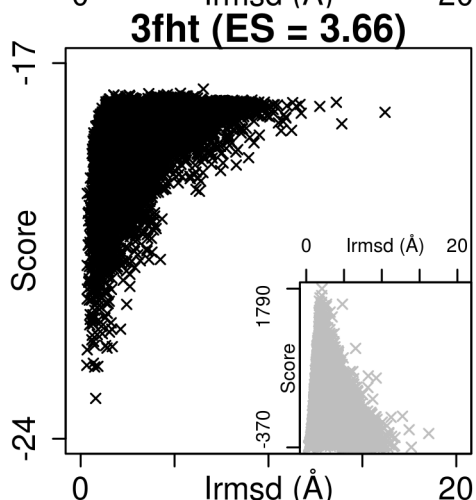

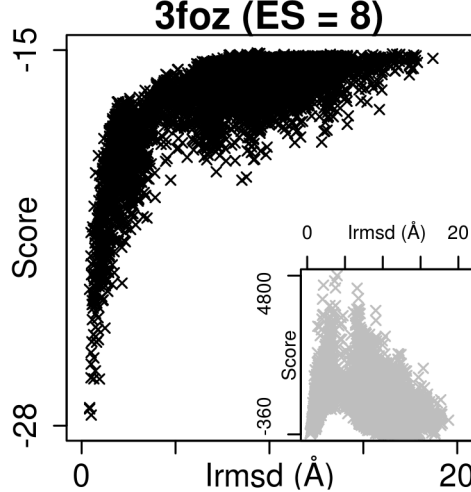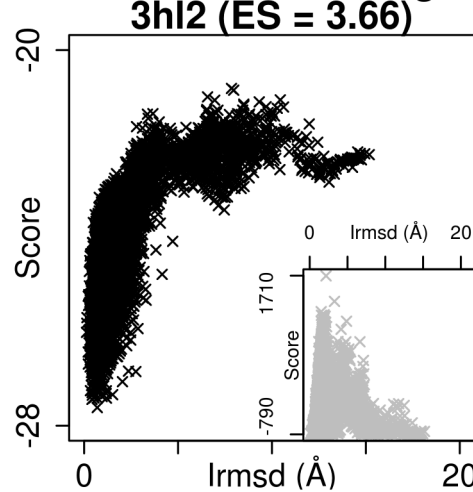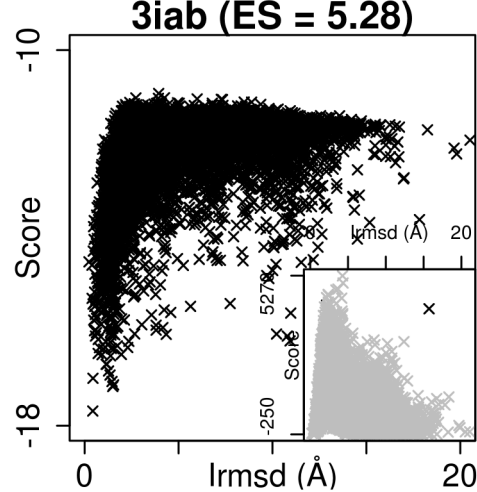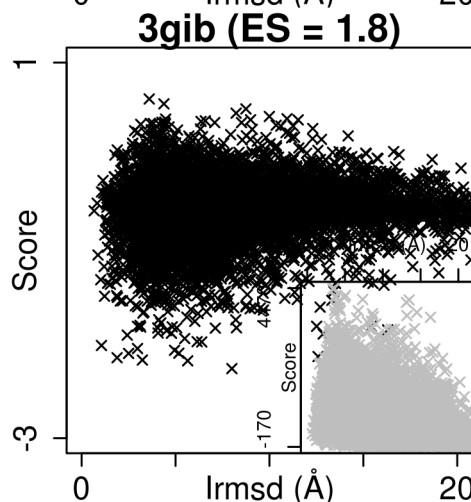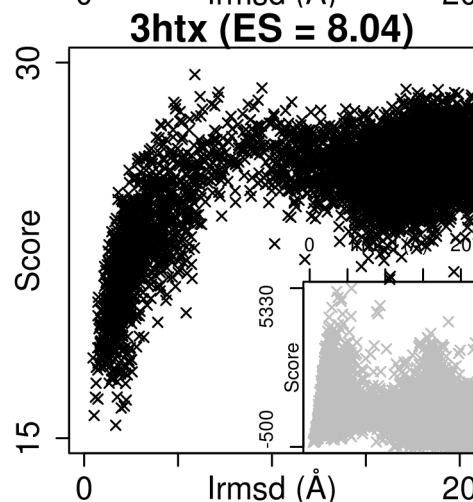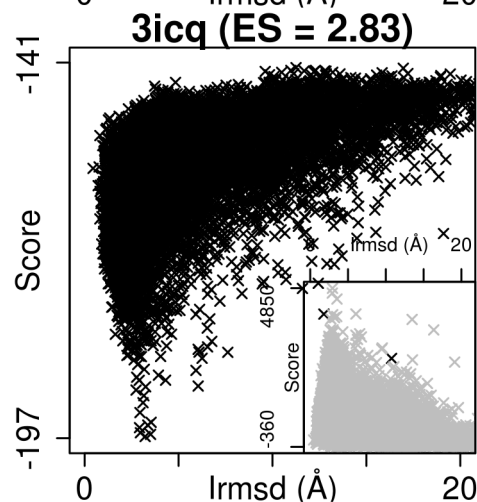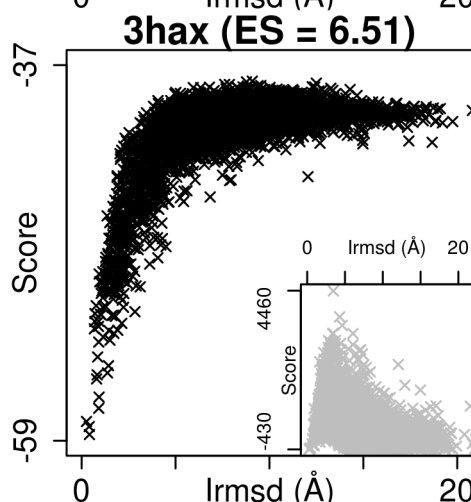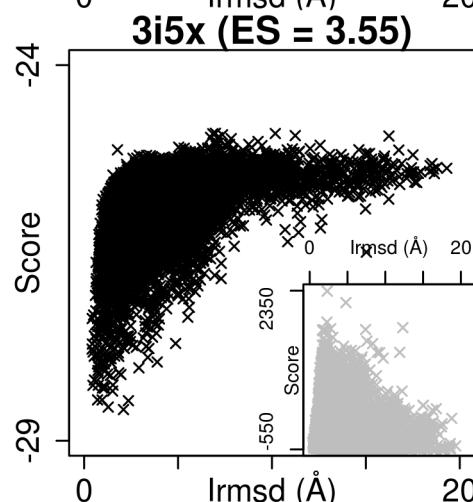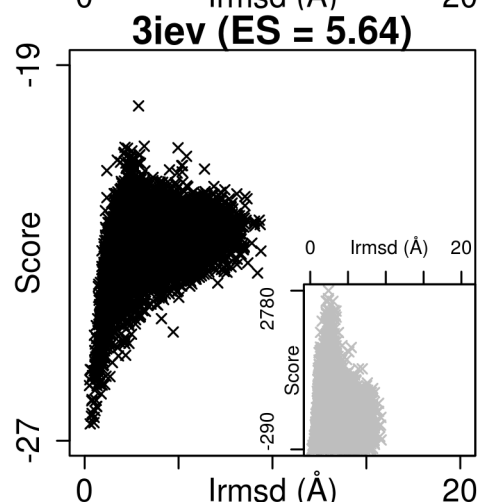

**3k62 (ES = 3.4)**

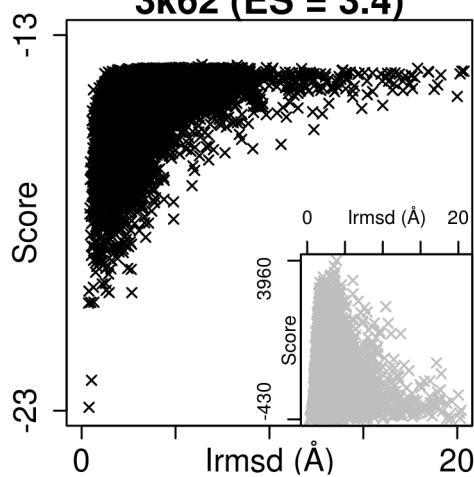

**3l25 (ES = 7.02)**

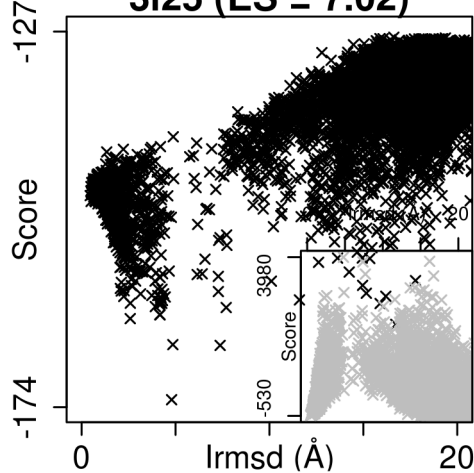

**3snp (ES = 6.63)**

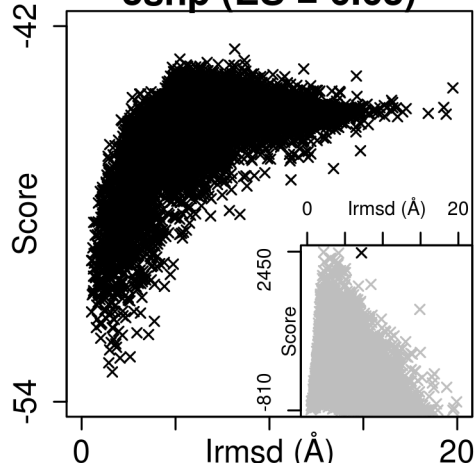

Supplement: Figure S2 — Energy vs Irmsd for the whole reference dataset in a leave-one-pdb-out setting. (PDF) [file pone.0108928.s002.pdf]

**1m5o (ES = 4.75)**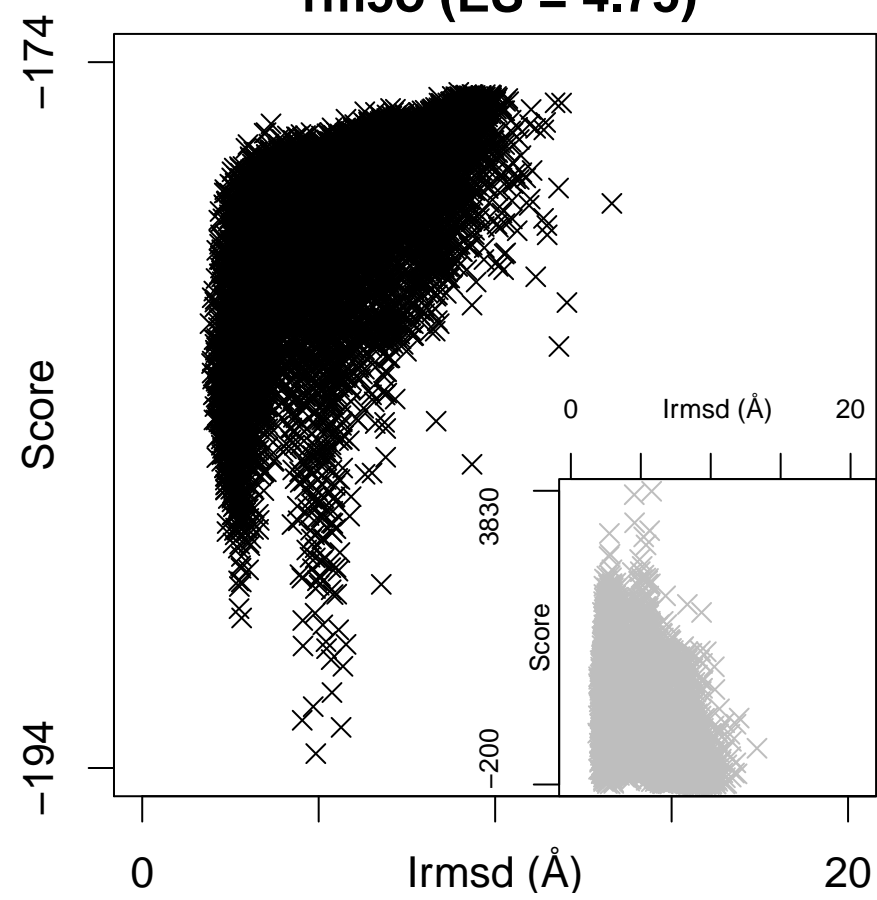**1qtq (ES = 6.09)**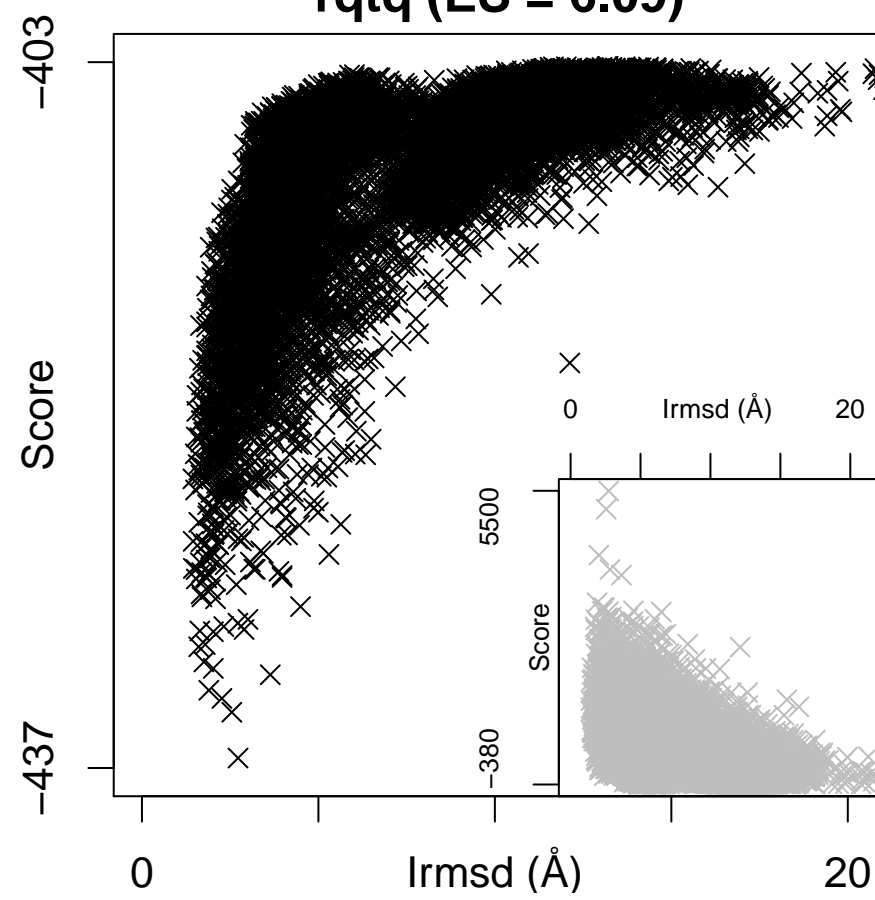**1wpu (ES = 3.15)**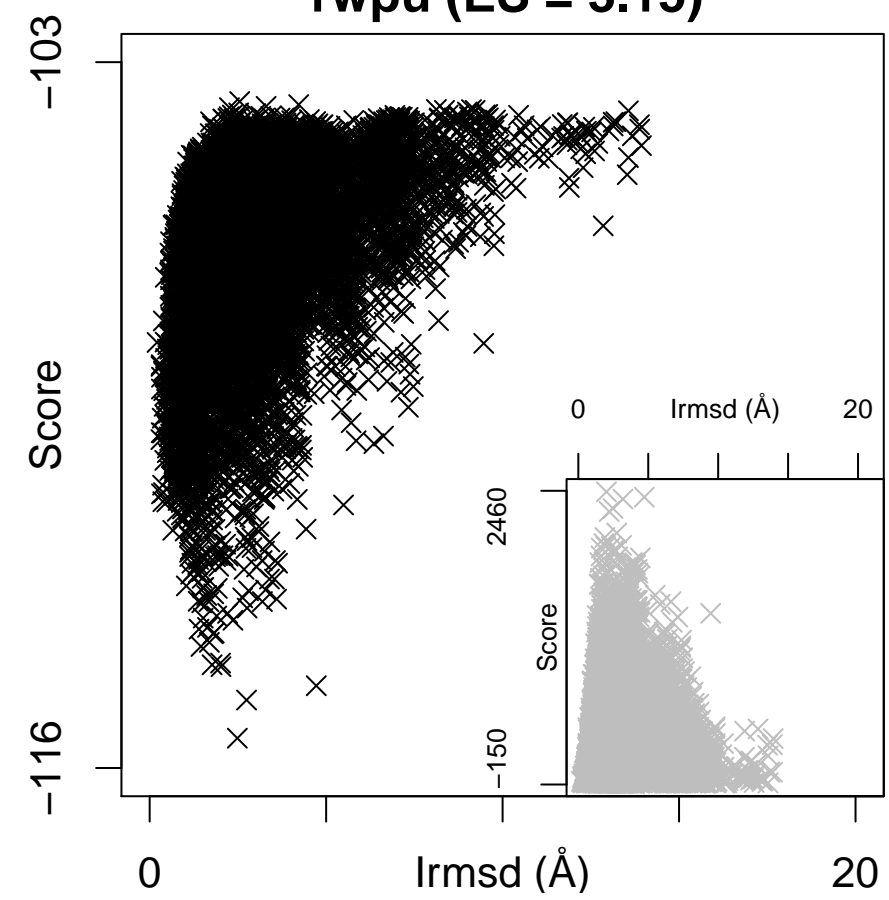**1yvp (ES = 0.2)**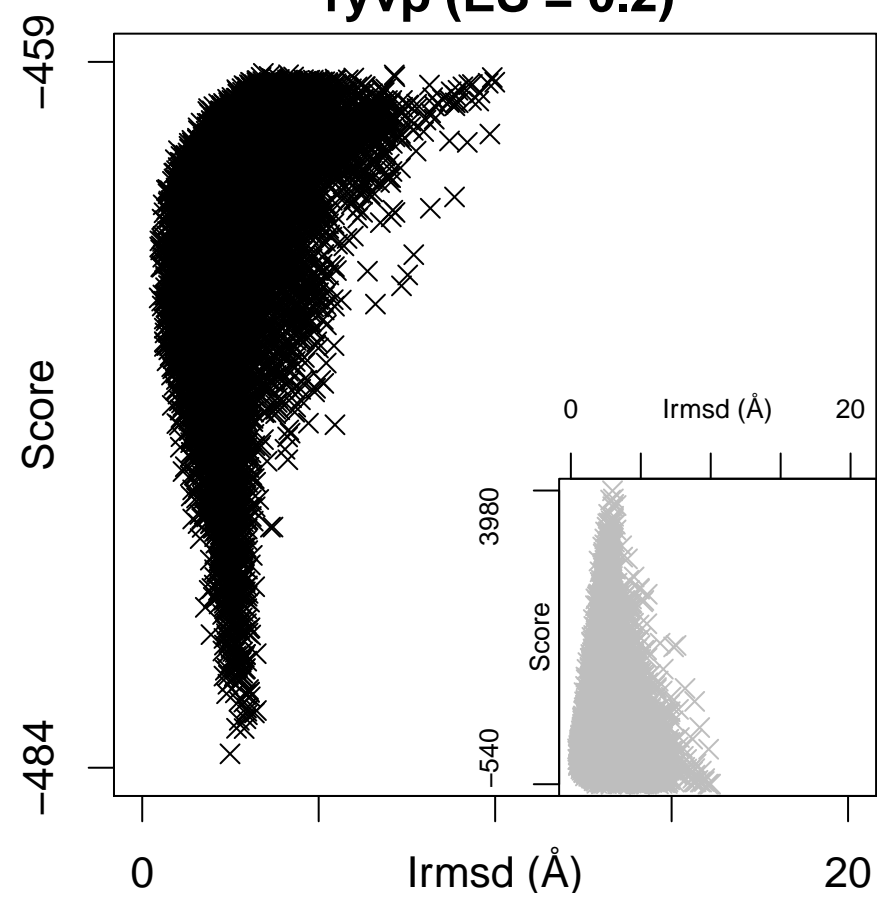**1zbh (ES = 0)**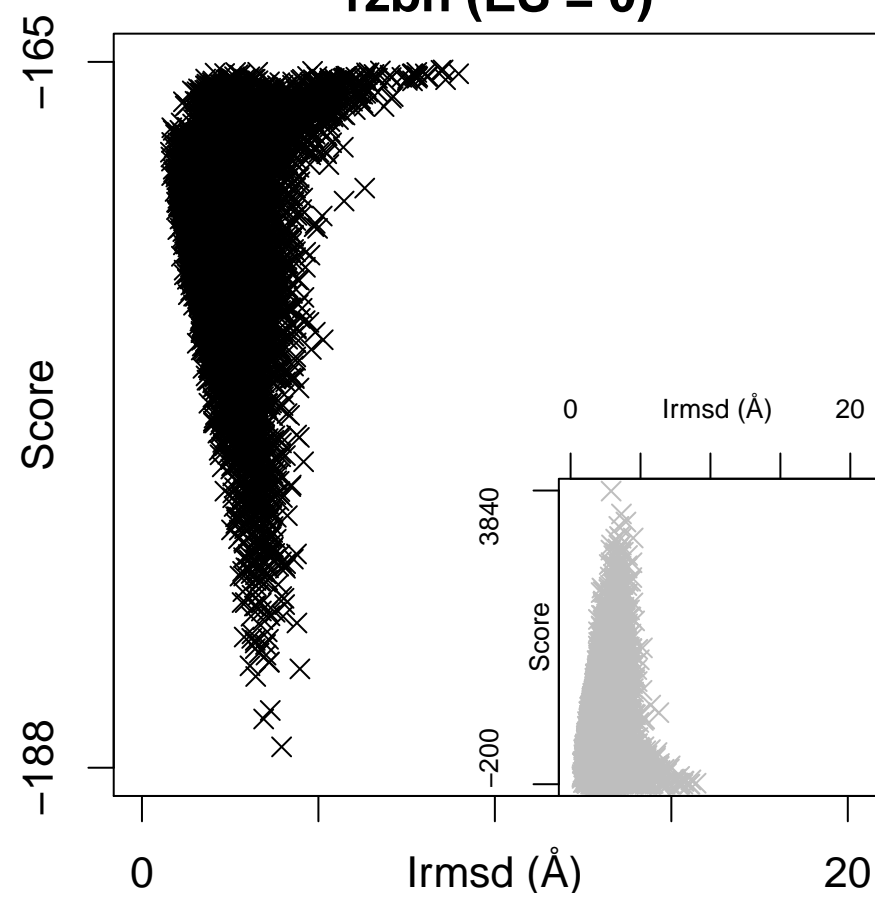**2ad9 (ES = 6.06)**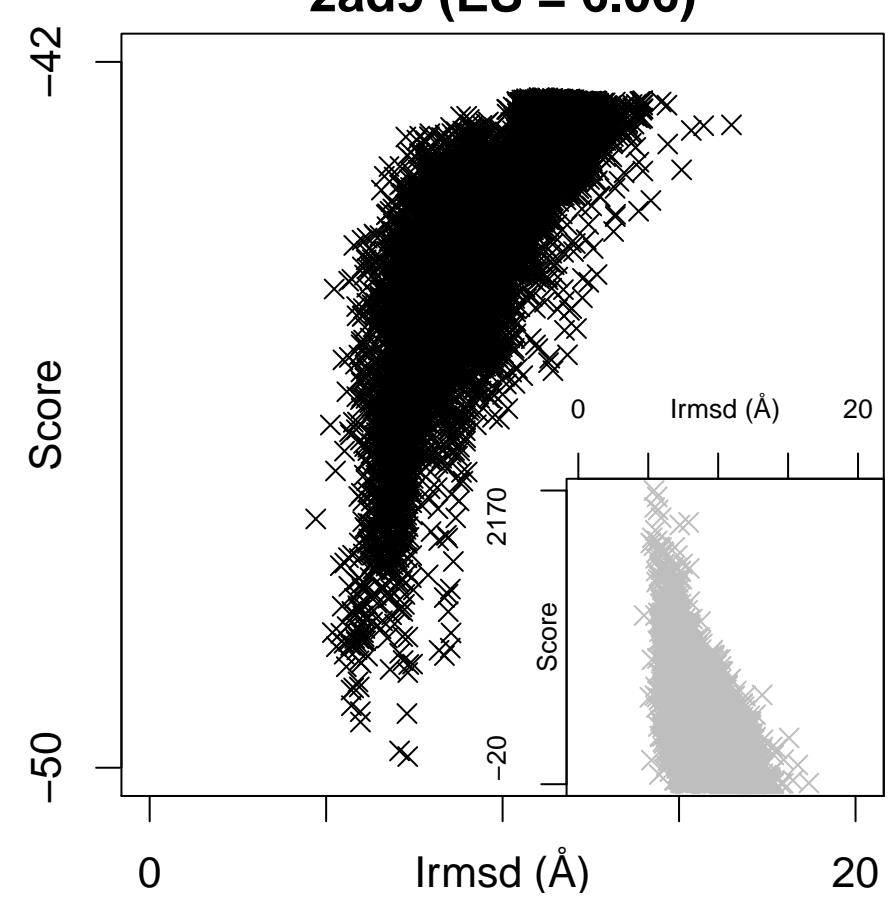

Supplement: Figure S4 — Energy vs Irmsd for the unbound test set. The 10,000 conformations evaluated for our optimized Rosetta scoring function are shown in black. On each plot, the bottom left panel show the equivalent non-optimized Rosetta result. (PDF) [file pone.0108928.s004.pdf]

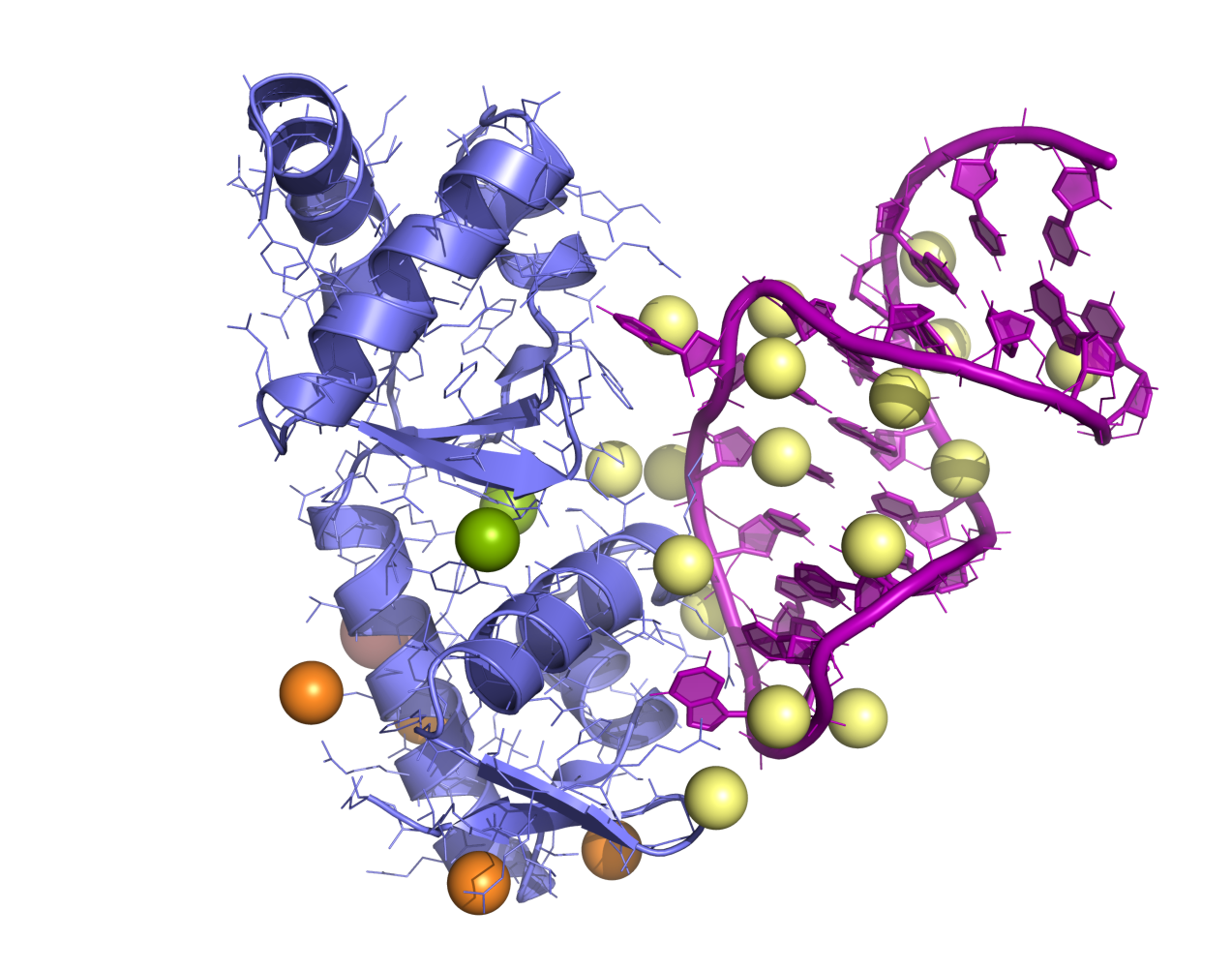

Supplement: Figure S5 — Structure of the mRNA binding domain of elongation factor SelB from E.coli in complex with SECIS RNA (PDB code 2pjp). Mg2+ ions (shown in yellow) are located at the interface and mediate the interaction. (TIFF) [file pone.0108928.s005.tiff]
