# Supplementary material for: Protein-RNA Complexes and Efficient Automatic Docking: Expanding RosettaDock Possibilities
Source: PLoS One. 2014 Sep 30;9(9):e108928. doi: 10.1371/journal.pone.0108928 (PMC4182525; doi:10.1371/journal.pone.0108928)
Supplement: Figure S3 — Energy vs Irmsd for the benchmark set in a bound setting. The 10,000 conformations evaluated for our optimized Rosetta scoring function are shown in black. On each plot, the bottom left panel shows the equivalent non-optimized Rosetta result. (PDF) [file pone.0108928.s003.pdf]

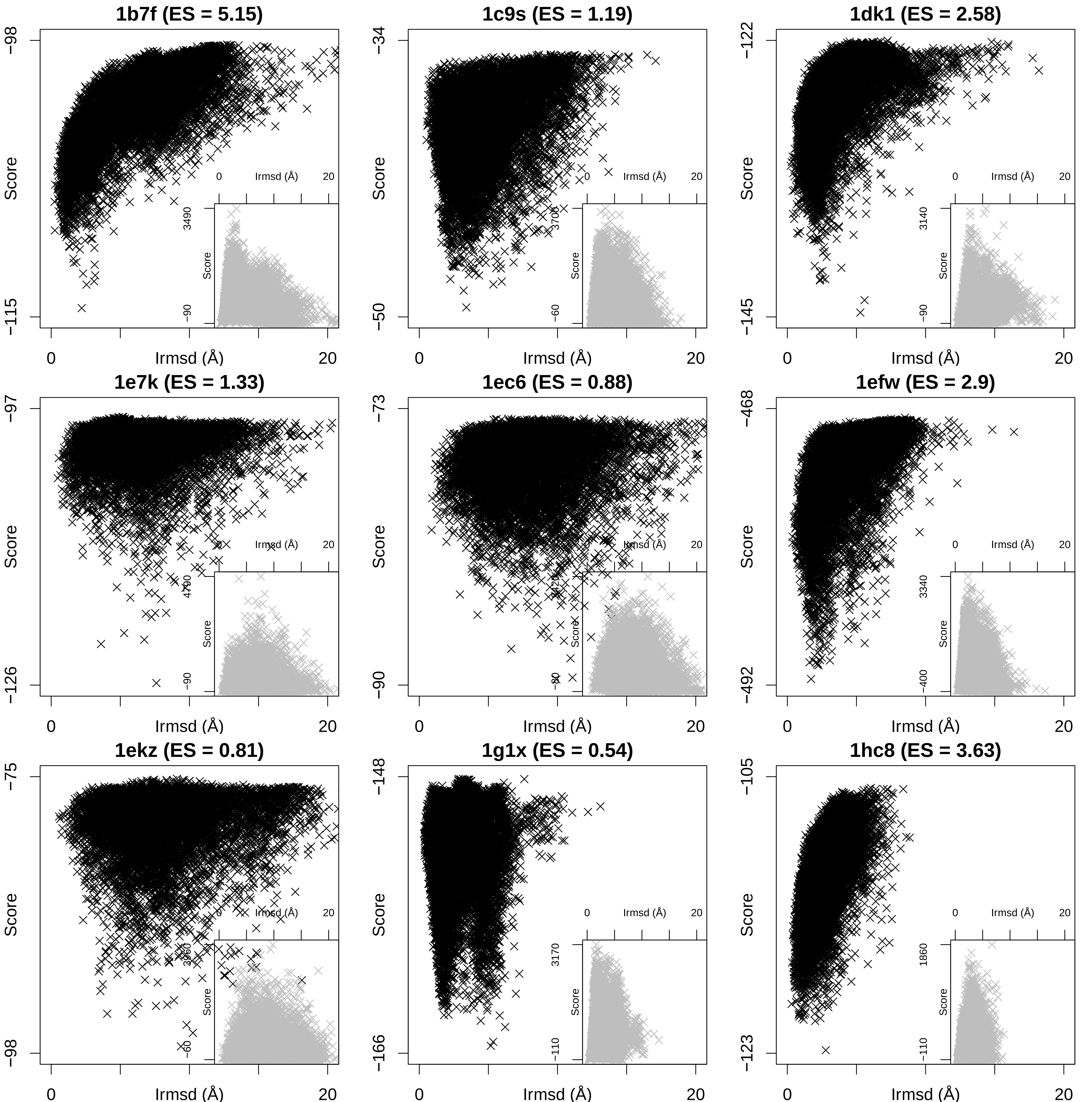
1b7f (ES = 5.15)
1c9s (ES = 1.19)
1dk1 (ES = 2.58)
1e7k (ES = 1.33)
1ec6 (ES = 0.88)
1efw (ES = 2.9)
1ekz (ES = 0.81)
1g1x (ES = 0.54)
1hc8 (ES = 3.63)
Score
Irmsd (Å)

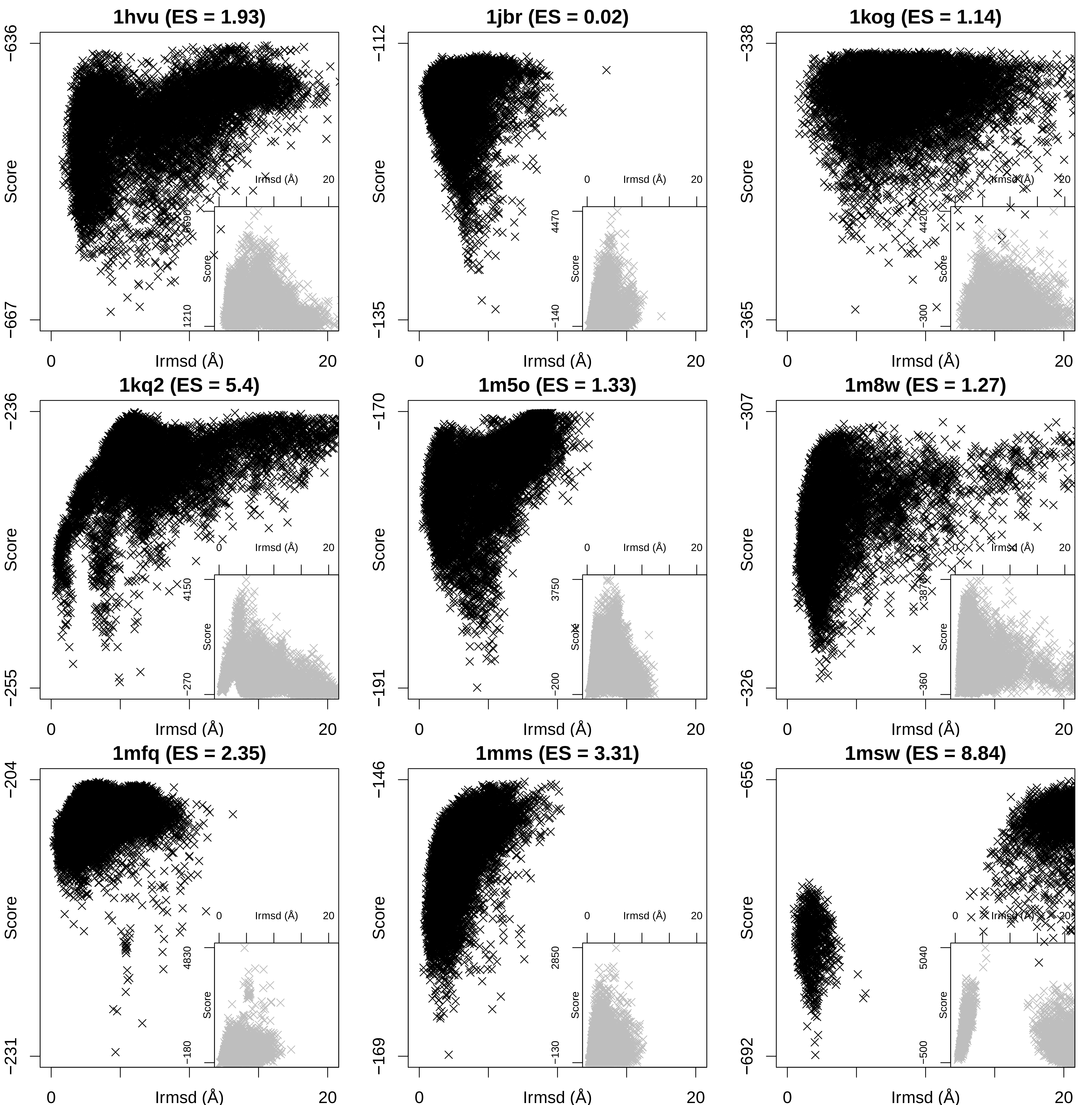
1hvu (ES = 1.93)
1jbr (ES = 0.02)
1kog (ES = 1.14)
1kq2 (ES = 5.4)
1m5o (ES = 1.33)
1m8w (ES = 1.27)
1mfq (ES = 2.35)
1mms (ES = 3.31)
1msw (ES = 8.84)
Score
Irmsd (Å)

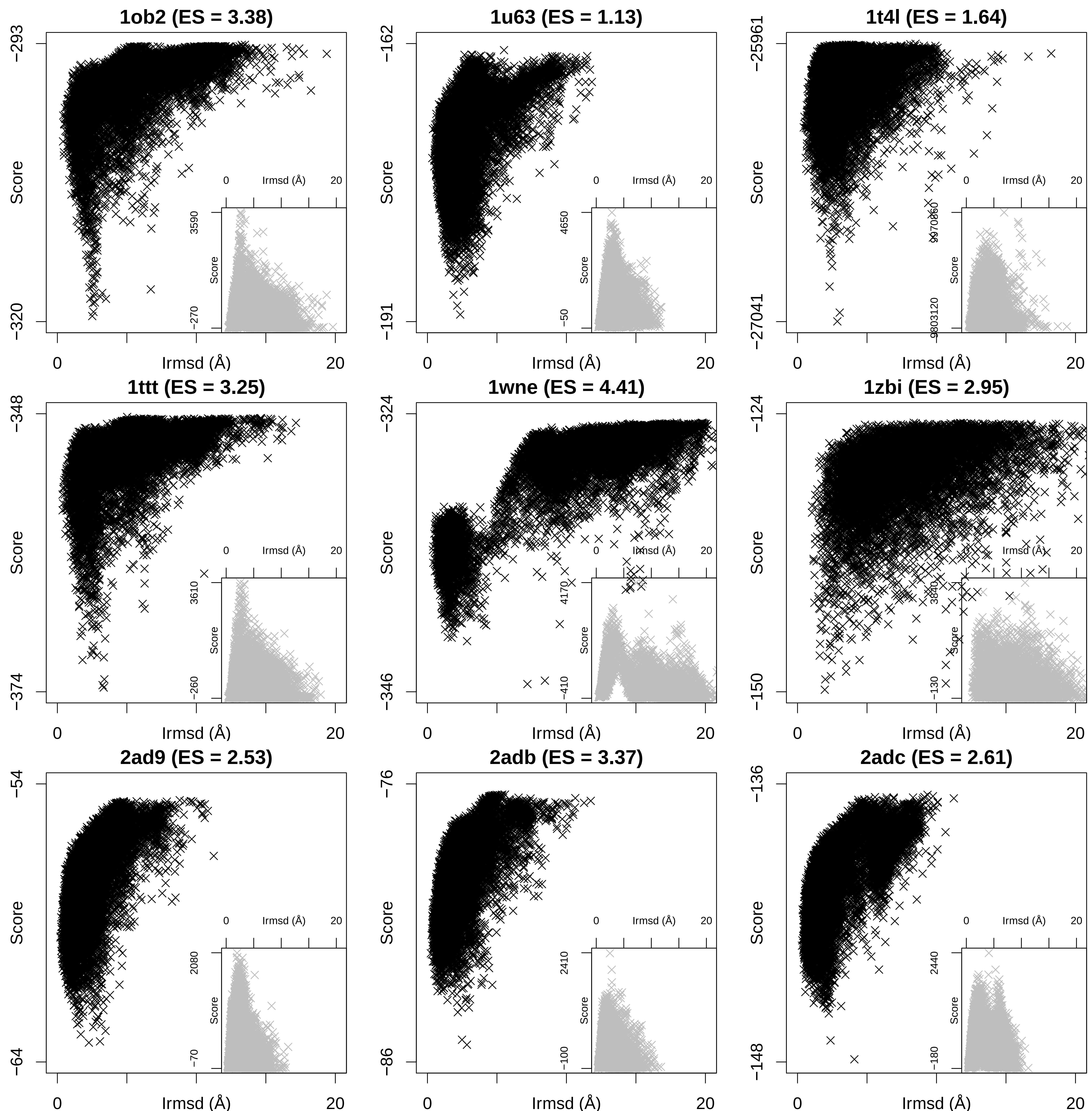

1ob2 (ES = 3.38)
1u63 (ES = 1.13)
1t4l (ES = 1.64)
1ttt (ES = 3.25)
1wne (ES = 4.41)
1zbi (ES = 2.95)
2ad9 (ES = 2.53)
2adb (ES = 3.37)
2adc (ES = 2.61)
Score
Irmsd (Å)
0
20
−293
−320
3590
−270
−162
−191
4650
−50
−25961
−27041
−348
−374
3610
−260
−324
−346
4170
−410
−124
−150
3840
−130
−54
−64
2080
−70
−76
−86
2410
−100
−136
−148
2440
−180

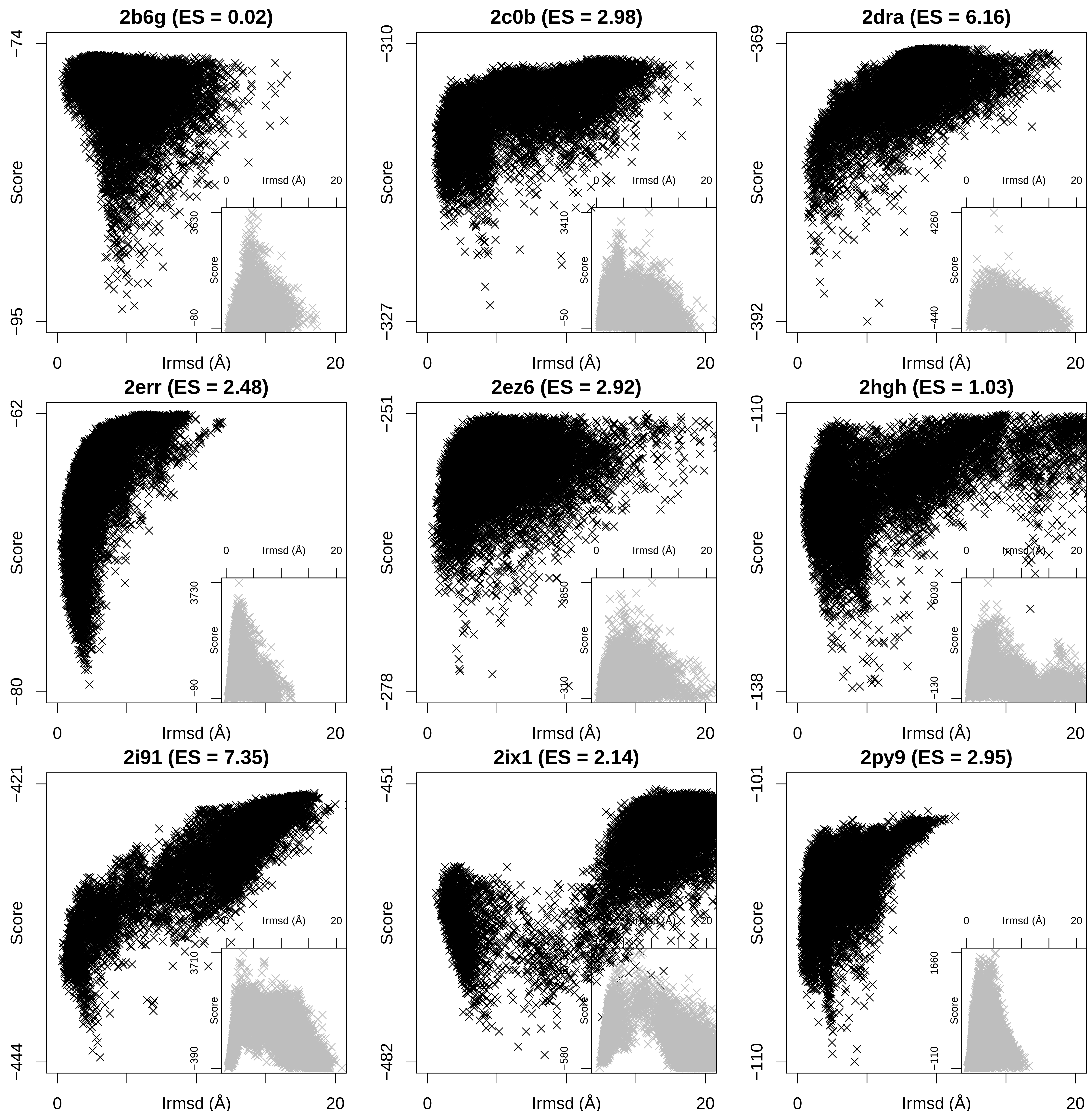

2b6g (ES = 0.02)
2c0b (ES = 2.98)
2dra (ES = 6.16)
2err (ES = 2.48)
2ez6 (ES = 2.92)
2hgh (ES = 1.03)
2i91 (ES = 7.35)
2ix1 (ES = 2.14)
2py9 (ES = 2.95)
Score
Irmsd (Å)
0
20
−74
−95
3630
−80
−310
−327
3410
−50
−369
−392
4260
−440
−62
−80
3730
−90
−251
−278
3850
−310
−110
−138
6030
−130
−421
−444
3710
−390
−451
−482
−580
−101
−110
1660
−110

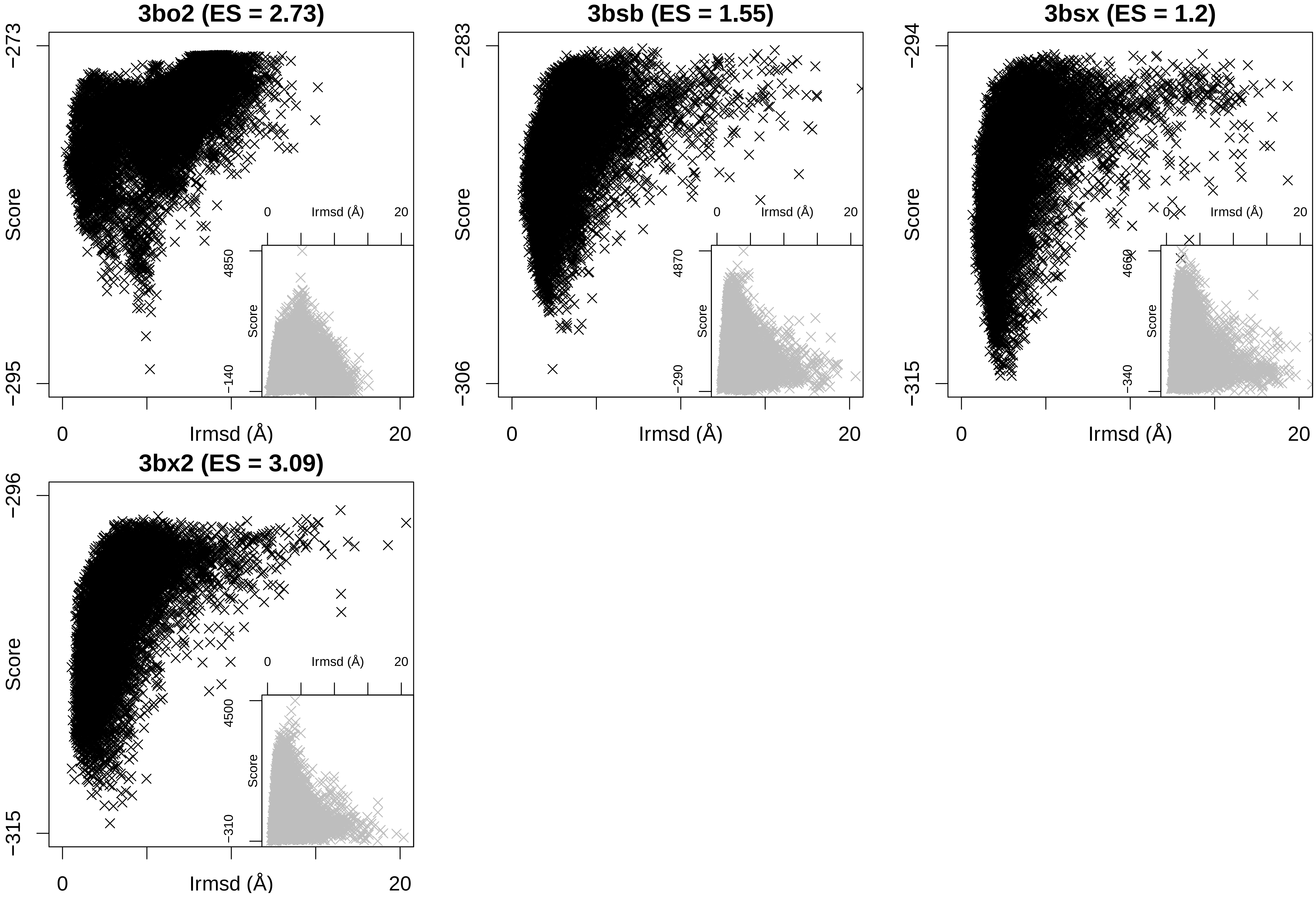
3bo2 (ES = 2.73)
−273
−295
Score
Irmsd (Å)
0
20
4850
−140
3bsb (ES = 1.55)
−283
−306
4870
−290
3bsx (ES = 1.2)
−294
−315
4660
−340
3bx2 (ES = 3.09)
−296
−315
4500
−310
