# Supplementary material for: Protein-RNA Complexes and Efficient Automatic Docking: Expanding RosettaDock Possibilities
Source: PLoS One. 2014 Sep 30;9(9):e108928. doi: 10.1371/journal.pone.0108928 (PMC4182525; doi:10.1371/journal.pone.0108928)
Supplement: Table S1 — Protein-RNA complexes reference set from the PRIDB. The rightmost column indicates putative redundancy with the docking benchmarks. The Type column refers to the structural family of the RNA molecule: single strand RNA (ssRNA), double strand RNA or single-stranded RNA of helical/paired structure (dsRNA) or transfer RNA (tRNA). (PDF) [file pone.0108928.s006.pdf]

PDB Code	Protein	# amino acids	RNA	# nucleic acids	Type	Also in benchmark
2zi0	Tomato aspermy virus protein 2b	60	siRNA	40	dsRNA	-
2gxb	H. sapiens dsRNA-specific adenosine deaminase	62	dsRNA 5'-UCGCGCG-3' and 5'-CGCGCG-3'	13	dsRNA	-
1hq1	E. coli signal recognition particle protein	76	4.5S RNA domain IV	49	dsRNA	-
2f8k	S. cerevisiae VTS1 protein SAM domain	84	5'-UAAUCUUUGACAGAUU-3'	16	dsRNA	II
1lng	M. jannaschii signal recognition particle 19 kDa protein	87	7S.S signal recognition particle RNA	97	dsRNA	-
3egz	H. sapiens U1 small nuclear ribonucleoprotein A	91	Tetracyclin haptamer and artificial riboswitch	65	dsRNA	-
1dfu	E. coli ribosomal protein L25	94	5S rRNA fragment	38	dsRNA	I & II
1jid	H. sapiens signal recognition particle 19 kDa protein	114	signal recognition particle RNA helix 6	29	dsRNA	-
2pjp	E. coli selenocysteine-specific elongation factor selB	121	SECIS RNA	23	dsRNA	-
1r9f	Tomato bushy stunt virus core protein 19	121	siRNA	40	dsRNA	-
2czj	T. thermophilus SsrA-binding protein	122	tmRNA tRNA domain	62	dsRNA	II
1wsu	M. thermoacetica selenocystein-specific elongation factor	124	5'-GGCGUUGCCGGUCGGCAACGCC-3'	22	dsRNA	I & II
2bu1	E. phage MS2 coat protein	129	5'-CAUGAGGAUUACCCAUG-3'	17	dsRNA	II
1di2	X. laevis protein A dsRNA-binding domain	129	dsRNA 5'-GGCGCGCGCC-3' tetramer	40	dsRNA	-
2zko	Influenza A virus non-structural protein 1 (NS1)	140	dsRNA	42	dsRNA	-
2az0	Flock house virus B2 protein	141	dsRNA 5'-GCAUGGACGCGUCCAUGC-3' dimer	36	dsRNA	I & II
1jbs	A. restrictus restrictocin	142	29-mer sarcin/ricin domain RNA analog	29	dsRNA	I
1un6	X. laevis transcription factor IIIA	145	5S rRNA fragment	61	dsRNA	-
1e8o	H. sapiens signal recognition particle 9 kDa and 14 kDa protein	149	7SL RNA	49	dsRNA	-
2anr	H. sapiens neuro-oncological ventral antigen 1 (NOVA 1)	155	5'-CUCGCGGAUCAGUCACCCAAGCGAG-3'	25	dsRNA	-
1feu	T. thermophilus 50S ribosomal protein L25	185	5S rRNA fragment	40	dsRNA	-
2i82	E. coli ribosomal large subunit pseudouridine synthase A	217	5'-GAGGGGAAUGAAAAUCCCCUC-3'	21	dsRNA	-
1mzp	S. acidocaldarius 50S ribosomal protein L1P	217	23S rRNA fragment	55	dsRNA	-
1zbh	H. sapiens 3'-5' exonuclease ERI1	224	histone mRNA stem-loop	16	dsRNA	I
2hw8	T. thermophilus 50S ribosomal protein L1	228	mRNA	36	dsRNA	I & II
2qux	P. phage coat protein	244	dsRNA	25	dsRNA	II

3iab	S. cerevisiae ribonuclease P/MRP protein subunit POP6 and POP7	255	Ribonuclease MRP RNA component P3 domain	46	dsRNA	-
3eqt	H. sapiens ATP-dependent RNA helicase DHX58	269	5'-GCGCGCGC-3'	8	dsRNA	-
3dd2	H. sapiens thrombine	288	dsRNA	26	dsRNA	-
2b3j	S. aureus tRNA adenosine deaminase (tAdA)	302	tRNA-ARG2 anticodon stem-loop	15	dsRNA	-
1k8w	E. coli tRNA-PseudoU synthase B	304	T stem-loop RNA	22	dsRNA	I & II
1r3e	T. maritima tRNA-PseudoU synthase B	305	tRNA-PseudoU T-arm	51	dsRNA	I & II
1ooa	M. musculus nuclear factor NF-kappa-B p105 subunit	313	RNA aptamer	29	dsRNA	-
1vfg	A. aeolicus tRNA nucleotidyltransferase	342	Primer tRNA	31	dsRNA	-
2ozb	H. sapiens U4/U6 small nuclear ribonucleoprotein Prp31	365	U4snRNA 5' stem-loop	33	dsRNA	-
3bt7	E. coli tRNA (uracile-5)-methyltransferase	369	19 nucleotide T-arm analogue	19	dsRNA	-
2bgg	A. fulgidus piwi AF1318 protein	395	dsRNA 5'-UUCGACGC-3' and 5'-GUCGAAUU-3'	16	dsRNA	I & II
2r8s	M. musculus synthetic FAB	433	P4-P6 RNA ribozyme domain	159	dsRNA	-
2nug	A. aeolicus ribonuclease III	434	dsRNA	44	dsRNA	-
1t0k	E. coli maltose-binding periplasmic-protein and S. cerevisiae 60S ribosomal protein L30	462	mRNA 3' consensus 5'-UGACC-3'	27	dsRNA	II
2e9t	Foot-and-mouth disease virus RNA-dependent RNA polymerase	474	dsRNA 5'-UAGGGCCC-3' and 5'-GGGCCCU-3'	15	dsRNA	-
3bso	Norwalk virus RNA-dependent RNA polymerase	479	primer-template RNA	16	dsRNA	II
3l25	Ebola virus polymerase cofactor VP35	492	dsRNA 5'-CGCAUGCG-3' dimer	16	dsRNA	-
1yvp	X. laevis 60 kDa SS-A Ro ribonucleoprotein	529	I & II strands of the Y RNA sequence	20	dsRNA	I & II
2w2h	Equine infectious anemia virus protein TAT and E. caballus cyclin-T1	581	TAR RNA	44	dsRNA	-
2gjw	A. fulgidus tRNA-splicing endonuclease	612	dsRNA	37	dsRNA	I & II
3ciy	M. musculus toll-like receptor 3	661	dsRNA	92	dsRNA	II
1q2r	Z. mobilis queunine tRNA ribosyltransferase	748	dsRNA 5'-AGCACGGCUNUAAACCGUGC-3' dimer	40	dsRNA	II
3htx	A. thaliana RNA methyltransferase HEN1	780	dsRNA	44	dsRNA	-
3snp	O. cuniculus cytoplasmic aconitate hydratase	850	Ferritin H IRE RNA	30	dsRNA	-
1tfw	A. fulgidus tRNA nucleotidyltransferase	874	Immature tRNA	26	dsRNA	-
3icq	S. cerevisiae GTP-binding nuclear protein GSP1/CNR1 and S. pombe exportin-T	1116	dsRNA	62	dsRNA	-
3a6p	H. sapiens exportin-5	1242	pre-miRNA	46	dsRNA	-
1n35	M. orthoreovirus minor core protein lambda 3	1264	dsRNA 5'-GGGGG-3' and 5'-UAGCCCCC-3'	13	dsRNA	I

2f8s	A. aeolicus argonaute protein	1408	dsRNA 5'-AGACAGCAUAUAUGCUGUCUUU-3' dimer	44	dsRNA	I
1m8v	P. abyssi putative snRNP sm-like protein	72	5'-UUUUUU-3'	6	ssRNA	I & II
1sds	M. jannaschii 50S ribosomal protein L7Ae	112	Archeal box H/ACA sRNA K-turn	15	ssRNA	I
2a8v	E. coli rho-dependent transcription termination factor RNA-binding domain	118	5'-CCC-3'	3	ssRNA	I
1si3	H. sapiens eukaryotic translation initiation factor 2C 1	120	5'-CGUGACUCU-3'	9	ssRNA	-
3d2s	H. sapiens musculeblind-like protein 1 (MBNL1)	135	5'-GCUGU-3'	5	ssRNA	-
1gtf	G. staerothermophilus TRP RNA-binding attenuation protein	142	5'-GAGU-3'	4	ssRNA	-
1wpu	B. subtilis hut operon positive regulatory protein	148	5'-UUGAGUU-3'	7	ssRNA	I & II
1fxl	P. encephalomyelitis antigen hud	167	5'-UUUUAUUUU-3'	9	ssRNA	-
2voo	H. sapiens lupus LA protein	179	5'-UUU-3'	3	ssRNA	-
3gib	E. coli protein Hfq	191	5'-AAAAAAAAA-3'	9	ssRNA	-
2asb	M. tuberculosis transcription elongation protein nusA	226	5'-GAACUCAAUAG-3'	11	ssRNA	I & II
1av6	Vaccinia cap-specific mRNA methyltransferase VP39	290	M7G Capped RNA 5'-GAAAAA-3'	7	ssRNA	-
1knz	S. rotavirus nonstructural RNA-binding protein 34	292	mRNA 3' consensus 5'-UGACC-3'	5	ssRNA	-
2gje	T. brucei mRNA-binding protein	292	Guide-RNA fragment	18	ssRNA	II
3iev	A. aeolicus GTP-binding protein	302	16S rRNA 3' end	11	ssRNA	-
1m8x	H. sapiens pumilio 1 homology domain	341	5'-UUGUAUAU-3'	8	ssRNA	-
2wj8	Human respiratory syncytial virus nucleoprotein	374	5'-CCCCCCC-3'	7	ssRNA	-
3fht	H. sapiens ATP-dependent RNA helicase DDX19B	392	5'-UUUUUU-3'	6	ssRNA	-
3k62	C. elegans Fem-3 mRNA-binding factor 2	400	5'-UGUGUUAUC-3'	9	ssRNA	-
2gtt	Rabies virus nucleoprotein	401	ssRNA	17	ssRNA	-
2gic	Vesicular stomatitis Indiana virus nucleocapsid protein	416	ssRNA fragment	15	ssRNA	I & II
2bh2	E. coli 23S ribosomal RNA (uracil-5)-methyltransferase RUMA	419	23S rRNA fragment	29	ssRNA	I & II
2jlv	Dengue virus serine protease subunit NS3	451	5'-AGACUAA-3'	7	ssRNA	-
2bx2	E. coli ribonuclease E	500	5'-UUUACAGUAUUUGU-3'	14	ssRNA	-
2po1	P. abyssi probable exosome complex exonuclease	503	5'-AAAAAAA-3'	7	ssRNA	-
3i5x	S. cerevisiae ATP-dependent RNA helicase MSS116	509	5'-UUUUUUUUUU-3'	10	ssRNA	-
1ddl	Desmodium yellow mottle tymovirus	551	5'-UUUUUUU-3'	7	ssRNA	-
1pgl	Bean pod mottle virus	555	5'-AGUCUC-3'	6	ssRNA	-
1uvj	P. phage P2 protein	664	5'-UUCC-3'	4	ssRNA	-
3ex7	H. sapiens CASC3 and mago nashi homolog and RNA-binding protein 8 and eukaryotic initiation factor 4A-III	687	5'-UUUUUU-3'	6	ssRNA	-

2vnu	S. cerevisiae exosome complex exonuclease RRP44	694	5'-AAAAAAAAA-3'	9	ssRNA	-
2z2q	Flock house viruse capsid protein	980	Flock house virus genomic RNA	12	ssRNA	-
2r7r	Simian rotavirus RNA-dependent RNA polymerase	1073	5'-UGUGACC-3'	7	ssRNA	II
1j1u	M. jannaschii tRNA-TYR synthetase	300	tRNA-TYR	74	tRNA	I & II
3foz	E. coli isopentenyl-tRNA transferase	303	tRNA-PHE	69	tRNA	-
2fk6	B. subtilis ribonuclease Z	307	tRNA-THR	53	tRNA	I & II
2fmt	E. coli tRNA-FMET formyltransferase	314	Formyl-methionyl-tRNA-FMET2	77	tRNA	I & II
2zzm	M. jannaschii uncharacterized protein MJ0883	329	tRNA-LEU	84	tRNA	-
2der	E. coli tRNA-specific 2-thiouridylase mnmA	348	tRNA-GLU	74	tRNA	-
3eph	S. cerevisiae tRNA isopentenyltransferase	402	tRNA	69	tRNA	-
1b23	T. aquaticus elongation factor EF-	405	E. coli tRNA-CYS	74	tRNA	II
1h3e	T. thermophilus tRNA-TYR synthetase	427	Wild type tRNA-TYR (GUA)	84	tRNA	II
1u0b	E. coli tRNA-CYS	461	tRNA-CYS	74	tRNA	I & II
2ct8	A. aeolicus tRNA-MET synthetase	465	tRNA-MET	74	tRNA	-
1n78	T. thermophilus tRNA-GLU synthetase	468	tRNA-GLU	75	tRNA	I & II
2d6f	M. thermautotrophicus tRNA-GLN amidotransferase	485	tRNA-GLN	72	tRNA	-
1asy	Yeast tRNA-ASP synthetase	490	tRNA-ASP	75	tRNA	I & II
3hax	P. furiosus probable tRNA-PseudoU synthase B and ribosome biogenesis protein Nop10 and 50S ribosomal protein L7Ae	503	H/ACA RNA	74	tRNA	-
2nqp	E. coli tRNA-PseudoU synthase A	528	tRNA-LEU	71	tRNA	-
1qtq	E. coli tRNA-GLN synthetase	529	tRNA-GLN II	74	tRNA	I & II
2zni	D. hafniense tRNA-PYL synthetase	558	tRNA-PYL	72	tRNA	-
1c0a	E. coli tRNA-ASP synthetase	585	tRNA-ASP	77	tRNA	I & II
1f7u	S. cerevisiae tRNA-ARG synthetase	607	tRNA-ARG	76	tRNA	I & II
2zue	P. horikoshii tRNA-ARG synthetase	628	tRNA-ARG	76	tRNA	-
1qf6	E. coli tRNA-THR synthetase	641	tRNA-THR	76	tRNA	I
2azx	H. sapiens tRNA-TRP synthetase	778	tRNA-TRP	72	tRNA	I & II
1ser	T. thermophilus tRNA-SER synthetase	793	tRNA-SER	65	tRNA	I & II
2bte	T. thermophilus aminoacyl-tRNA synthetase	877	tRNA-LEU transcript with anticodon CAG	78	tRNA	I & II
3hl2	H. sapiens O-phosphoseryl-tRNA-SEC selenium transferase	886	tRNA-SEC	82	tRNA	-
1ffy	S. aureus tRNA-ILE synthetase	917	tRNA-ILE	75	tRNA	-
1h4s	T. thermophilus tRNA-PRO synthetase	946	tRNA-PRO (CGG)	67	tRNA	I & II
1wz2	P. horikoshii tRNA-LEU synthetase	948	tRNA-LEU	88	tRNA	-
2du3	A. fulgidus O-phosphoseryl-tRNA synthetase	1001	tRNA-CYS	71	tRNA	-
2iy5	T. thermophilus tRNA-PHE synthetase	1117	tRNA-PHE	76	tRNA	II(1eiy)
1j2b	P. horikoshii archaeosine tRNA-GUA transglycosylase	1152	tRNA-VAL	77	tRNA	II
