# Supplementary material for: Protein-RNA Complexes and Efficient Automatic Docking: Expanding RosettaDock Possibilities
Source: PLoS One. 2014 Sep 30;9(9):e108928. doi: 10.1371/journal.pone.0108928 (PMC4182525; doi:10.1371/journal.pone.0108928)
Supplement: Table S2 — Protein-RNA complexes for the test set. For each complex the unbound column for protein and RNA reports the PDB code of the unbound structures when available. The difficulty codes are taken from [36], [38]. (PDF) [file pone.0108928.s007.pdf]

PDB Code	Originating Benchmark	Difficulty	Unbound protein	Description	# amino acids	Unbound RNA	RNA description	# nucleic acids	Type
2b6g	I	R	2d3d	S. cerevisiae VTS1 protein SAM domain	81	2b7g	5'-GGAGGCUCUGGCAGCUUUC-3'	19	dsRNA
1zbi	I	R	1zbf	B. halodurans Ribonuclease H-related protein	135	-	RNA-DNA hydrid	24	dsRNA
2ez6	I	R	1jfz	A. aeolicus ribonuclease III	218	-	dsRNA	56	dsRNA
1ttt	I	R	1eft	T. aquaticus elongation factor EF-TU	405	4tna	Yeast tRNA-PHE	76	tRNA
1msw	I	X	1aro	E. phage DNA-directed T7 RNA polymerase	863	-	mRNA	47	ssRNA
1ec6	II	R	1dtj	H. sapiens neuro-oncological ventral antigen 2 (NOVA 2)	87	-	dsRNA	20	dsRNA
1t4l	II	R	1t4o	S. cerevisiae ribonuclease III	90	-	snRNA 47 precursor 5' terminal hairpin	32	dsRNA
3bo2	II	R	1nu4	H. sapiens U1 small nuclear ribonucleoprotein A	95	-	Group I intron P9 and mRNA	221	dsRNA
1g1x	II	R	1ris	T. thermophilus 30S ribosomal protein S6	98	-	16S rRNA fragment	84	dsRNA
1jbr	II	R	1aqz	A. restrictus restrictocin	149	-	31-mer SRD RNA analog	31	dsRNA
1u63	II	R	1l2a	M. jannaschii 50S ribosomal protein L1P	214	-	mRNA	49	dsRNA
1kog	II	R	1evl	E. coli tRNA-THR synthetase	401	-	tRNA-THR synthetase mRNA operator essential domain	37	dsRNA
1c9s	II	R	1qaw	G. staerothermopilus TRP RNA-binding attenuation protein	67	-	5'-GAUGAGA-3'	7	ssRNA
3bsb	II	R	1m8z	H. sapiens pumilio 1 homology domain	341	-	5'-UUUAAUGUU-3'	9	ssRNA
3bsx	II	R	1m8z	H. sapiens pumilio 1 homology domain	341	-	5'-UUGUAAUAUU-3'	10	ssRNA

1kq2	II	R	1kq1	S. aureus host factor protein for Q beta	365	-	5'-AUUUUUG-3'	7	ssRNA
2i91	II	R	1yvr	S. laevis 60 kDa SS-A Ro ribonucleoprotein	526	-	misfolded RNA fragment	23	ssRNA
1efw	II	R	1l0w	T. thermophilus tRNA-ASP synthetase	580	1c0a	tRNA-ASP	73	tRNA
1hc8	II	S	1foy	G. staerothermopilus ribosomal protein L11	74	-	23S rRNA fragment	57	dsRNA
1dk1	II	S	2fkx	T. thermophilus 30S ribosomal protein S15	86	-	30S rRNA fragment	57	dsRNA
1mfq	II	S	1qb2	H. sapiens signal recognition particle 54 kDa protein	108	1l9a	7S RNA of Human signal recognition particle	128	dsRNA
1mms	II	S	2k3f	T. maritima ribosomal protein L11	133	-	23S rRNA fragment	58	dsRNA
2err	II	S	2cq3	H. sapiens ataxin-2-binding protein 1 (Fox 1)	88	-	5'-UGCAUGU-3'	7	ssRNA
2ad9	II	S	1sjq	H. sapiens polypyrimidine tract-binding protein RBD1	98	-	5'-CUCUCU-3'	6	ssRNA
2adb	II	S	1sjr	H. sapiens polypyrimidine tract-binding protein RBD2	127	-	5'-CUCUCU-3'	6	ssRNA
2py9	II	S	2jzx	H. sapiens poly(rC)-binding protein 2	141	-	Human telomeric RNA	12	ssRNA
2adc	II	S	2evz	H. sapiens polypyrimidine tract-binding protein RBD3 and 4	208	-	5'-CUCUCU-3'	6	ssRNA
3bx2	II	S	1m8z	H. sapiens pumilio 1 homology domain	328	-	5'-UGUAUAUUA-3'	9	ssRNA
1ekz	II	X	1stu	D. melanogaster maternal effect protein	76	-	RNA hairpin	30	dsRNA
2hgh	II	X	2j7j	S. laevis transcription factor IIIA	87	-	5S rRNA fragment	55	dsRNA
1hvu	II	X	2vg5	Human immunodeficiency virus HIV1 reverse transcriptase	954	-	RNA pseudoknot	30	dsRNA

1b7f	II	X	3sxl	D. melanogaster SXL-lethal protein	167	-	5'-GUUGUUUUUUUU-3'	12	ssRNA
2c0b	II	X	2vmk	E. coli ribonuclease E	488	-	5'-UUUACAGUAUU-3'	11	ssRNA
1ob2	II	X	1efc	E. coli elongation factor EF-TU	393	1ehz	tRNA-PHE	76	tRNA
1m5o	II (&I)	R	1nu4	H. sapiens U1 small nuclear ribonucleoprotein A	92	-	RNA substrate and RNA hairpin ribozyme	113	dsRNA
1wne	II (&I)	R	1u09	Foot-and-mouth disease virus RNA-dependent RNA polymerase	476	-	dsRNA 5'-CAUGGGCC-3' and 5'-GGCCC-3' dimer	13	dsRNA
1m8w	II (&I)	R	1mz8	H. sapiens pumilio 1 homology domain	340	-	5'-UUGUAUAU-3'	8	ssRNA
2ix1	II (&I)	R	2id0	E. coli exoribonuclease II	643	-	5'-AAAAAAAAAAAAA-3'	13	ssRNA
1e7k	II (&I)	S	2jnb	H. sapiens 15.5 kDa RNA-binding protein	125	-	5'-GCCAAUGAGGCCGAGGC-3'	17	dsRNA
2dra	II (&I as 2drb)	R	1r89	A. fulgidus CCA-adding enzyme	437	1vfg	tRNA mini DCC	34	dsRNA
