# Supplementary material for: Protein-RNA Complexes and Efficient Automatic Docking: Expanding RosettaDock Possibilities
Source: PLoS One. 2014 Sep 30;9(9):e108928. doi: 10.1371/journal.pone.0108928 (PMC4182525; doi:10.1371/journal.pone.0108928)
Supplement: Table S3 — Leave-one-pdb-out scoring statistics for the reference dataset. Enrichment Score, 10 best energy candidates, 100 best energy candidates, number of near-native structures and Area Under the ROC Curve are reported for each native structure both using the non-optimized RosettaDock scoring function (Default) and our optimized scoring function (ROGER). (PDF) [file pone.0108928.s008.pdf]

PDB code	Enrichment		Top10			Top100		# near native	AUC	
	Default	Roger	Default	Roger	Expected	Default	Roger		Default	Roger
1asy	0.49	3.69	7	8	7.195	28	95	7195	42.95%	74.00%
1av6	3.15	1.53	5	10	8.815	77	100	8815	38.17%	88.26%
1b23	1.47	2.87	4	10	5.037	4	99	5037	39.38%	81.24%
1c0a	0.46	5.89	1	10	2.568	11	99	2568	30.41%	91.29%
1ddl	0.52	2.64	2	3	2.789	18	35	2789	47.79%	64.22%
1dfu	2.11	5.07	4	10	9.240	47	100	9240	27.89%	93.34%
1di2	0.59	4.05	10	10	7.553	99	98	7553	40.52%	90.53%
1e8o	1.18	2.08	6	2	3.376	24	37	3376	44.09%	62.81%
1f7u	0.17	4.46	4	10	4.105	4	94	4105	20.72%	88.76%
1feu	1.16	1.72	4	10	9.081	65	99	9081	33.69%	81.13%
1ffy	0.02	7.09	0	10	3.121	0	100	3121	42.32%	93.05%
1fxl	0.08	6.26	0	10	5.104	0	100	5104	17.98%	78.67%
1gtf	4.28	1.61	10	10	9.628	97	100	9628	49.39%	71.97%
1h3e	0.41	5.02	0	10	5.918	11	100	5918	35.43%	76.62%
1h4s	0.81	1.90	1	4	1.190	2	34	1190	41.88%	62.91%
1hq1	1.43	2.42	6	3	2.624	31	40	2624	57.27%	67.35%
1j1u	1.56	1.40	10	9	8.331	96	92	8331	53.03%	66.67%
1j2b	0.02	6.25	0	10	1.317	0	100	1317	13.26%	86.67%
1jbs	0.58	0.79	0	0	2.528	1	5	2528	44.14%	60.90%
1jid	2.52	0.05	2	10	9.357	63	98	9357	37.07%	65.01%
1k8w	0.59	7.73	1	10	3.916	5	100	3916	35.28%	93.78%
1knz	0.00	5.68	0	10	3.110	0	100	3110	14.91%	92.51%
1lng	2.74	3.91	6	10	7.299	70	95	7299	60.99%	65.15%
1m8v	0.85	1.70	10	3	7.017	90	51	7017	43.97%	53.79%
1m8x	1.68	4.61	7	10	8.413	86	100	8413	42.64%	75.23%
1mzp	0.76	3.15	0	6	1.592	11	51	1592	40.37%	71.41%
1n35	0.10	7.27	0	8	0.732	0	85	732	33.80%	98.59%
1n78	0.33	3.67	0	10	4.390	0	98	4390	29.73%	92.34%
1ooa	3.45	2.56	6	10	8.662	75	100	8662	43.74%	90.53%
1pgl	3.13	0.00	10	9	9.344	80	98	9344	24.48%	85.09%
1q2r	0.00	6.33	0	10	4.585	0	100	4585	23.71%	81.80%
1qf6	0.09	7.30	0	10	1.154	0	99	1154	23.09%	90.18%
1qtq	0.05	6.36	0	10	2.971	4	100	2971	30.37%	89.09%
1r3e	0.66	6.89	0	10	3.356	10	100	3356	40.54%	90.50%
1r9f	1.55	3.19	10	10	9.359	99	100	9359	38.47%	87.11%
1sds	0.80	1.51	5	6	4.183	41	37	4183	55.21%	58.58%
1ser	0.10	4.35	0	9	2.999	0	95	2999	29.18%	95.64%
1si3	0.27	4.66	0	10	7.681	0	100	7681	7.99%	75.39%
1t0k	1.53	1.34	8	0	3.725	59	14	3725	55.28%	51.03%
1tfw	0.02	4.38	0	10	0.225	0	97	225	22.32%	99.91%
1u0b	0.02	6.33	0	10	2.616	0	97	2616	21.30%	87.02%
1un6	0.66	2.93	0	9	3.952	1	93	3952	29.64%	83.37%
1uvj	0.51	3.20	0	6	2.066	0	73	2066	31.46%	79.98%
1vfg	2.96	0.32	9	10	9.313	87	99	9313	41.66%	73.83%
1wpu	2.16	3.48	10	10	8.746	78	100	8746	51.50%	62.11%
1wsu	0.50	1.72	0	2	4.816	14	32	4816	36.42%	52.40%
1wz2	0.25	5.18	0	10	2.451	0	98	2451	31.08%	79.24%

1yvp	2.26	2.69	8	10	9.384	84	100	9384	37.24%	84.41%
1zbh	0.15	1.74	2	8	3.809	4	69	3809	34.27%	67.79%
2a8v	0.14	2.20	2	8	4.775	14	84	4775	33.93%	53.05%
2anr	0.55	2.06	7	0	2.927	23	18	2927	38.11%	57.12%
2asb	1.18	5.26	2	10	5.475	3	100	5475	45.69%	92.22%
2az0	0.22	4.33	0	10	1.351	1	77	1351	39.63%	84.06%
2azx	0.13	2.29	0	9	7.220	10	90	7220	28.63%	63.42%
2b3j	1.57	3.18	0	10	7.172	7	100	7172	24.43%	92.68%
2bgg	0.30	4.89	0	10	5.420	4	100	5420	27.60%	87.89%
2bh2	0.29	7.08	0	10	2.340	0	100	2340	32.26%	88.99%
2bte	0.98	2.15	7	10	7.251	76	98	7251	42.13%	81.78%
2bu1	0.18	2.41	5	3	3.909	17	54	3909	38.21%	60.05%
2bx2	0.07	2.08	0	5	3.441	0	42	3441	36.48%	81.18%
2ct8	0.68	3.09	3	10	5.817	37	87	5817	35.47%	79.30%
2czj	3.12	3.51	10	10	9.135	94	100	9135	51.17%	77.64%
2d6f	2.30	2.75	10	10	8.458	81	96	8458	62.86%	66.99%
2der	0.88	4.52	0	10	3.535	0	100	3535	26.04%	96.07%
2du3	2.14	2.11	9	6	9.160	65	84	9160	47.78%	51.75%
2e9t	0.00	7.70	0	10	1.688	0	100	1688	19.00%	99.80%
2f8k	1.37	1.47	9	1	4.993	78	32	4993	50.85%	56.92%
2f8s	1.27	2.19	6	10	6.802	53	100	6802	40.24%	66.73%
2fk6	0.84	2.93	10	10	8.170	78	98	8170	46.25%	85.70%
2fmt	0.59	5.99	0	10	2.675	10	100	2675	39.56%	89.93%
2gic	1.11	3.06	0	8	3.956	0	87	3956	37.72%	91.87%
2gje	1.11	2.60	6	9	7.233	74	95	7233	30.27%	82.87%
2gjw	0.11	4.25	8	10	7.806	28	100	7806	21.62%	89.19%
2gtt	1.03	3.38	0	9	2.419	0	88	2419	43.48%	87.67%
2gxb	0.54	1.87	0	3	4.715	16	45	4715	49.49%	59.28%
2hw8	1.89	3.72	9	10	7.302	23	99	7302	52.29%	92.75%
2i82	3.45	7.14	6	10	6.908	60	100	6908	40.69%	84.12%
2iy5	0.07	6.69	0	4	0.920	0	79	920	30.81%	95.72%
2jlv	0.53	0.74	0	1	4.519	0	13	4519	33.33%	81.37%
2nqp	0.74	3.02	0	5	2.642	5	58	2642	43.36%	74.03%
2nug	0.29	6.56	0	10	0.365	0	100	365	66.33%	100.00%
2ozb	1.10	6.82	1	10	3.716	3	100	3716	37.17%	84.30%
2pjp	0.01	1.75	1	1	2.983	1	33	2983	31.18%	59.71%
2po1	1.98	1.35	10	10	9.139	97	100	9139	44.74%	75.15%
2qux	0.16	3.18	1	4	1.940	1	56	1940	39.84%	70.33%
2r7r	0.03	4.54	0	10	4.231	0	98	4231	17.52%	90.99%
2r8s	1.35	1.96	8	10	7.263	42	90	7263	45.88%	58.69%
2vnu	0.09	2.20	0	2	2.481	0	58	2481	24.02%	92.64%
2voo	0.08	1.69	10	10	7.556	96	100	7556	42.96%	84.91%
2w2h	0.50	3.96	2	10	5.472	20	99	5472	35.93%	81.34%
2wj8	0.06	6.11	0	10	2.429	0	99	2429	21.94%	91.47%
2z2q	0.49	2.64	3	9	5.908	26	89	5908	41.04%	60.01%
2zi0	3.01	0.48	0	9	7.917	10	88	7917	26.80%	79.77%
2zko	1.15	4.04	0	7	1.338	9	83	1338	56.67%	82.85%
2zni	0.30	6.44	0	10	2.671	0	100	2671	39.96%	75.52%
2zue	0.30	4.53	0	10	4.929	0	99	4929	22.06%	89.45%
2zzm	0.11	8.06	0	10	1.256	0	100	1256	17.74%	93.10%

3a6p	6.30	7.17	0	7	1.972	54	96	1972	80.50%	98.57%
3bso	0.94	5.84	0	10	2.472	1	100	2472	43.48%	99.16%
3bt7	0.57	4.98	1	10	5.376	20	100	5376	32.53%	78.92%
3ciy	1.71	2.79	4	10	5.629	44	82	5629	59.08%	68.67%
3d2s	0.04	0.79	6	10	8.929	62	97	8929	31.56%	71.09%
3dd2	0.74	2.00	0	3	2.468	4	43	2468	44.36%	65.72%
3egz	1.05	3.05	6	10	5.223	6	97	5223	16.40%	78.69%
3eph	0.19	8.81	0	10	1.002	0	100	1002	13.09%	99.27%
3eqt	0.40	7.19	2	10	5.188	54	100	5188	38.30%	94.21%
3ex7	0.05	4.06	0	10	7.516	0	96	7516	17.89%	74.52%
3fht	2.19	3.66	0	10	8.250	0	100	8250	17.70%	84.03%
3foz	0.48	8.00	0	10	1.341	0	100	1341	21.01%	98.21%
3gib	0.37	1.80	0	6	2.324	0	48	2324	30.84%	48.98%
3hax	0.04	6.51	1	10	1.027	1	100	1027	19.17%	87.71%
3hl2	2.67	3.66	0	10	9.062	56	100	9062	47.50%	98.70%
3htx	0.02	8.04	0	10	1.045	0	100	1045	22.19%	95.66%
3i5x	0.31	3.55	0	10	6.791	4	100	6791	35.47%	79.65%
3iab	0.59	5.28	0	10	4.770	7	92	4770	23.74%	73.43%
3icq	0.14	2.83	0	10	5.140	10	88	5140	34.96%	80.57%
3iev	1.30	5.64	8	10	7.914	67	100	7914	60.50%	65.06%
3k62	2.51	3.40	9	10	8.482	86	100	8482	35.14%	77.82%
3l25	0.35	7.02	0	7	1.205	0	79	1205	38.48%	98.58%
3snp	0.02	6.63	1	10	3.201	1	100	3201	25.46%	83.77%
