# Supplementary material for: Protein-RNA Complexes and Efficient Automatic Docking: Expanding RosettaDock Possibilities
Source: PLoS One. 2014 Sep 30;9(9):e108928. doi: 10.1371/journal.pone.0108928 (PMC4182525; doi:10.1371/journal.pone.0108928)
Supplement: Table S4 — Scoring results on the bound benchmark test set. Enrichment Score, 10 best energy candidates, 100 best energy candidates, number of near-native structures and Area Under the ROC Curve are reported for each native structure both using the non-optimized RosettaDock scoring function (Default) and our optimized scoring function (ROGER). (PDF) [file pone.0108928.s009.pdf]

PDB code	Enrichment score		Top10			Top100		# near native	AUC	
	Default	Roger	Default	Roger	Expected	Default	Roger		Default	Roger
1b7f	0.47	5.15	1	10	3.579	1	100	3579	0.32	0.89
1c9s	0.9	1.19	6	7	5.905	25	81	5905	0.37	0.69
1dk1	2.32	2.58	9	8	7.66	89	97	7660	0.61	0.82
1e7k	0.67	1.33	2	1	3.359	11	16	3359	0.50	0.53
1ec6	0.31	0.88	1	0	1.73	3	14	1730	0.44	0.60
1efw	0.41	2.9	0	10	6.366	0	96	6366	0.33	0.79
1ekz	0.95	0.81	4	1	1.768	18	20	1768	0.52	0.52
1g1x	3.32	0.54	2	6	8.764	85	82	8764	0.59	0.51
1hc8	1.56	3.63	10	10	9.433	94	100	9433	0.49	0.79
1hvu	0.77	1.93	2	2	4.366	42	48	4366	0.50	0.70
1jbr	3.56	0.02	9	9	9.229	71	86	9229	0.45	0.65
1kog	0.95	1.14	3	1	1.532	11	23	1532	0.49	0.54
1kq2	0.09	5.4	0	9	2.602	0	89	2602	0.31	0.74
1m5o	1.06	1.33	6	5	4.748	6	78	4748	0.24	0.83
1m8w	1.79	1.27	4	10	8.343	80	97	8343	0.37	0.74
1mfq	1.36	2.35	10	3	7.757	91	52	7757	0.62	0.59
1mms	2.52	3.31	10	9	8.859	85	96	8859	0.50	0.86
1msw	0.00	8.84	0	10	0.841	0	98	841	0.08	1.00
1ob2	0.78	3.38	7	10	4.625	12	97	4625	0.41	0.83
1t4l	0.56	1.64	10	10	7.852	98	95	7852	0.52	0.61
1ttt	0.76	3.25	6	10	4.691	11	97	4691	0.39	0.78
1u63	3.14	1.13	7	10	8.338	72	100	8338	0.44	0.86
1wne	0.01	4.41	0	7	2.213	0	96	2213	0.17	0.99
1zbi	0.37	2.95	0	8	2.231	0	69	2231	0.34	0.75
2ad9	1.99	2.53	10	10	9.179	84	100	9179	0.31	0.90
2adb	1.03	3.37	10	10	9.18	75	100	9180	0.25	0.89
2adc	3.38	2.61	3	10	6.94	40	100	6940	0.44	0.86
2b6g	1.73	0.02	9	6	5.228	93	62	5228	0.58	0.42
2c0b	0.00	2.98	0	8	3.937	0	91	3937	0.26	0.86
2dra	0.03	6.16	0	8	1.419	0	88	1419	0.32	0.91
2err	1.27	2.48	8	10	8.263	20	100	8263	0.15	0.92
2ez6	1.42	2.92	10	9	5.235	87	67	5235	0.58	0.62
2hgh	0.74	1.03	0	4	5.387	0	69	5387	0.25	0.82
2i91	0.47	7.35	0	10	1.726	0	95	1726	0.28	0.98
2ix1	0.02	2.14	0	4	1.795	0	55	1795	0.17	0.94
2py9	4.28	2.95	9	10	8.014	89	98	8014	0.50	0.83
3bo2	0.66	2.73	4	6	3.663	4	82	3663	0.31	0.80
3bsb	1.6	1.55	7	10	8.348	81	100	8348	0.36	0.80
3bsx	1.6	1.2	3	10	8.448	83	100	8448	0.32	0.82
3bx2	1.83	3.09	8	10	8.595	75	100	8595	0.33	0.84
